# Age-dependency of terminal ileum tissue resident memory T cell responsiveness profiles to *S*. Typhi following oral Ty21a immunization in humans

**DOI:** 10.1186/s12979-021-00227-y

**Published:** 2021-04-19

**Authors:** Jayaum S. Booth, Eric Goldberg, Seema A. Patil, Robin S. Barnes, Bruce D. Greenwald, Marcelo B. Sztein

**Affiliations:** 1 Center for Vaccine Development and Global Health, University of Maryland School of Medicine, Baltimore, MD 21201 USA; 2 Department of Pediatrics, University of Maryland School of Medicine, Baltimore, MD USA; 3 Department of Medicine, University of Maryland School of Medicine, Baltimore, MD USA; 4 Division of Gastroenterology and Hepatology, University of Maryland School of Medicine, Baltimore, MD USA; 5 Program in Oncology, University of Maryland Marlene and Stewart Greenebaum Comprehensive Cancer Center, Baltimore, MD 21201 USA

**Keywords:** Aging, Vaccine-induced responses, Tissue resident memory T cells, Terminal ileum LPMC, Ty21a, Oral vaccine

## Abstract

**Background:**

The impact of aging on the immune system is unequivocal and results in an altered immune status termed immunosenescence. In humans, the mechanisms of immunosenescence have been examined almost exclusively in blood. However, most immune cells are present in tissue compartments and exhibit differential cell (e.g., memory T cells -T_M_) subset distributions. Thus, it is crucial to understand immunosenescence in tissues, especially those that are exposed to pathogens (e.g., intestine). Using a human model of oral live attenuated typhoid vaccine, Ty21a, we investigated the effect of aging on terminal ileum (TI) tissue resident memory T (T_RM_) cells. T_RM_ provide immediate adaptive effector immune responsiveness at the infection site. However, it is unknown whether aging impacts T_RM_ *S*. Typhi-responsive cells at the site of infection (e.g., TI). Here, we determined the effect of aging on the induction of TI *S.* Typhi-responsive T_RM_ subsets elicited by Ty21a immunization.

**Results:**

We observed that aging impacts the frequencies of TI-lamina propria mononuclear cells (LPMC) T_M_ and T_RM_ in both Ty21a-vaccinated and control groups. In unvaccinated volunteers, the frequencies of LPMC CD103- CD4+ T_RM_ displayed a positive correlation with age whilst the CD4/CD8 ratio in LPMC displayed a negative correlation with age**.** We observed that elderly volunteers have weaker *S.* Typhi-specific mucosal immune responses following Ty21a immunization compared to adults. For example, CD103+ CD4+ T_RM_ showed reduced IL-17A production, while CD103- CD4+ T_RM_ exhibited lower levels of IL-17A and IL-2 in the elderly than in adults following Ty21a immunization. Similar results were observed in LPMC CD8+ T_RM_ and CD103- CD8+ T cell subsets. A comparison of multifunctional (MF) profiles of both CD4+ and CD8+ T_RM_ subsets between elderly and adults also showed significant differences in the quality and quantity of elicited single (S) and MF responses.

**Conclusions:**

Aging influences tissue resident T_M_ *S*. Typhi-specific responses in the terminal ileum following oral Ty21a-immunization. This study is the first to provide insights in the generation of local vaccine-specific responses in the elderly population and highlights the importance of evaluating tissue immune responses in the context of infection and aging.

**Trial registration:**

This study was approved by the Institutional Review Board and registered on ClinicalTrials.gov (*identifier* NCT03970304*, Registered 29 May 2019 - Retrospectively registered)*.

**Supplementary Information:**

The online version contains supplementary material available at 10.1186/s12979-021-00227-y.

## Background

Aging is associated with the waning of the immune system which progressively declines in function resulting in the diminution of humoral and cellular immune responses [1, 2], a process termed immunosenescence. Studies have shown that negative clinical outcomes in populations of older adults correlate with immunosenescence [3, 4]. For example, viral and bacterial infections (e.g., influenza, herpes zoster and pneumococcal diseases) are more severe in older adults than in younger adults [5–7]. While vaccines are among the most cost-effective interventions in public health against infectious diseases, vaccine-induced responses in the elderly are usually of lower magnitude and do not confer long-term protective immunity in this population [8–11]. Alterations in the T cell repertoire also have been characterized in aging [12, 13]. However, our current understanding of human immunosenescence is primarily derived from studies using peripheral blood while age-associated changes in the mucosal microenvironment are extremely limited. This represents a crucial knowledge gap as mucosal infections (e.g., respiratory, gastrointestinal and urinary tracts) are major causes of morbidity and mortality in the elderly. Furthermore, it is important to understand immunity to pathogens at the infection sites (mostly tissues) in the context of aging.

The induction of antigen-specific T cell and B cell responses are crucial for protective immunity against pathogens. In the elderly, both the quantity and quality of antibody responses are inferior to those observed among younger individuals [14]. Importantly, aging affects the T cell compartment as shown by the contraction of the naïve T cell repertoire [15] and expansion of terminally differentiated cell subsets with altered effector functions [16]. Recently, a new memory T cell subset abundant in peripheral tissues, tissue resident memory T cells (T_RM_) have been shown to be central for eliciting and mediating protective immunity at the site of infection [17, 18]. T_RM_ represent a non-migratory population of T_M_ that is phenotypically different from circulating T_M_ subsets (e.g., T central memory (T_CM_) and T effector memory (T_EM_)) and mediate rapid effector immune responses following antigen recall [18]. Human T_RM_ are mainly characterized phenotypically by high expression of CD69, a key marker for distinguishing between circulating and resident T_M_ [19]. Integrin αEβ7 (CD103), the ligand to E-cadherin, is also used to characterize T_RM_ but CD103 expression is mostly confined to CD8+ T_RM_ and a minor subset of CD4+ T_RM_ [19–22]. In the human intestine, the majority of CD4+ T_RM_ are CD103- CD69+ and a minority are CD103+ CD69+ [19] while CD8+ T_RM_ are mostly CD103+ CD69+. Very little information is available concerning the presence and role of either CD4+ and CD8+ T_RM_ populations during immunosenescence. It is also unclear whether these local T_RM_ cells are affected by the aging process in terms of quantity (frequencies of cell subsets) and, importantly, in the characteristics of their responses to pathogens at the site of infection following infection or oral immunization. Furthermore, we have a very limited understanding of the mechanisms of immunosenescence in the local intestinal mucosa.

Most human pathogens infect the host through mucosal sites; however, few licensed oral vaccines (e.g., *Salmonella enterica serovar* Typhi (*S*. Typhi)) are available. Currently, there are two licensed typhoid vaccines in the USA, namely Ty21a, a live attenuated oral vaccine and the parenteral Vi polysaccharide vaccine [23, 24]. Ty21a can invade the mucosa and replicate for only a few cycles, mimicking natural infection. Ty21a confers a moderate level of long-lived protection (60–80%, 5–7 years) in the field [24]. However, the mechanisms by which *S*. Typhi or Ty21a induce T_RM_ responses in the TI has not yet been fully explored in adults [25–30] and no information is available in the elderly. Although the human gastrointestinal tract constitutes a major reservoir of total body lymphocytes (~ 60%) and represents an area of high antigenic exposure, our understanding of the mechanisms of protection from infection and oral vaccination in the gut mucosa is very limited, particularly with respect to the immunologic events that follow the administration of oral vaccines. This wide gap in knowledge is impeding the rational development of new oral vaccines for pathogens that gain access to the host through the gastrointestinal tract, especially for the elderly.

In this study, we analyzed age-related changes of TI LPMC memory T cells obtained from Ty21a-vaccinated and unvaccinated individuals by comparing: (i) TI LPMC CD4+ and CD8+ T_M_/T_RM_ cell subsets frequencies, and (ii) TI LPMC CD4+ and CD8+ T_M_/T_RM_ *S*. Typhi-specific responses following Ty21a immunization. These comparisons provide unique insights into the generation of age-associated *S*. Typhi specific responses in the human TI mucosa.

## Results

### Aging influences the frequencies of TI LPMC T_M_ and T_RM_ cells subsets

Recent evidence suggests that aging influences the frequencies and absolute numbers of T cells in peripheral blood. However, the effect of aging on T cells frequencies in the human intestine particularly in the terminal ileum (TI) is unknown. To explore this phenomenon, we characterized freshly isolated TI-LPMC CD4+ and CD8+ T cells including their memory subset distribution, as well as tissue resident subsets in specimens obtained from biopsies of Ty21a-vaccinated and unvaccinated volunteers using the gating strategy depicted in Fig. S1A. No differences in total LPMC T cell frequencies as measured by CD3 expression were detected between elderly (≥ 60 yrs.) and adults (< 60 yrs.) regardless of Ty21a immunization (Fig. S1B). Interestingly, CD3+ CD4+ T cell frequencies were observed to decrease significantly (*p* < 0.05) in elderly as compared to adult unvaccinated volunteers. However following Ty21a, no differences were observed in the frequencies of CD4+ T cells (Fig. S1B). Regarding CD3+ CD8+ T cells, we observed a trend (*p* = 0.08) to be present at higher levels in the elderly than in adult unvaccinated volunteers (Fig. S1B). Similar to CD4+ T cells, no differences were observed in the frequencies of CD8+ T cells following Ty21a immunization (Fig. S1B).

**Fig. 1:**
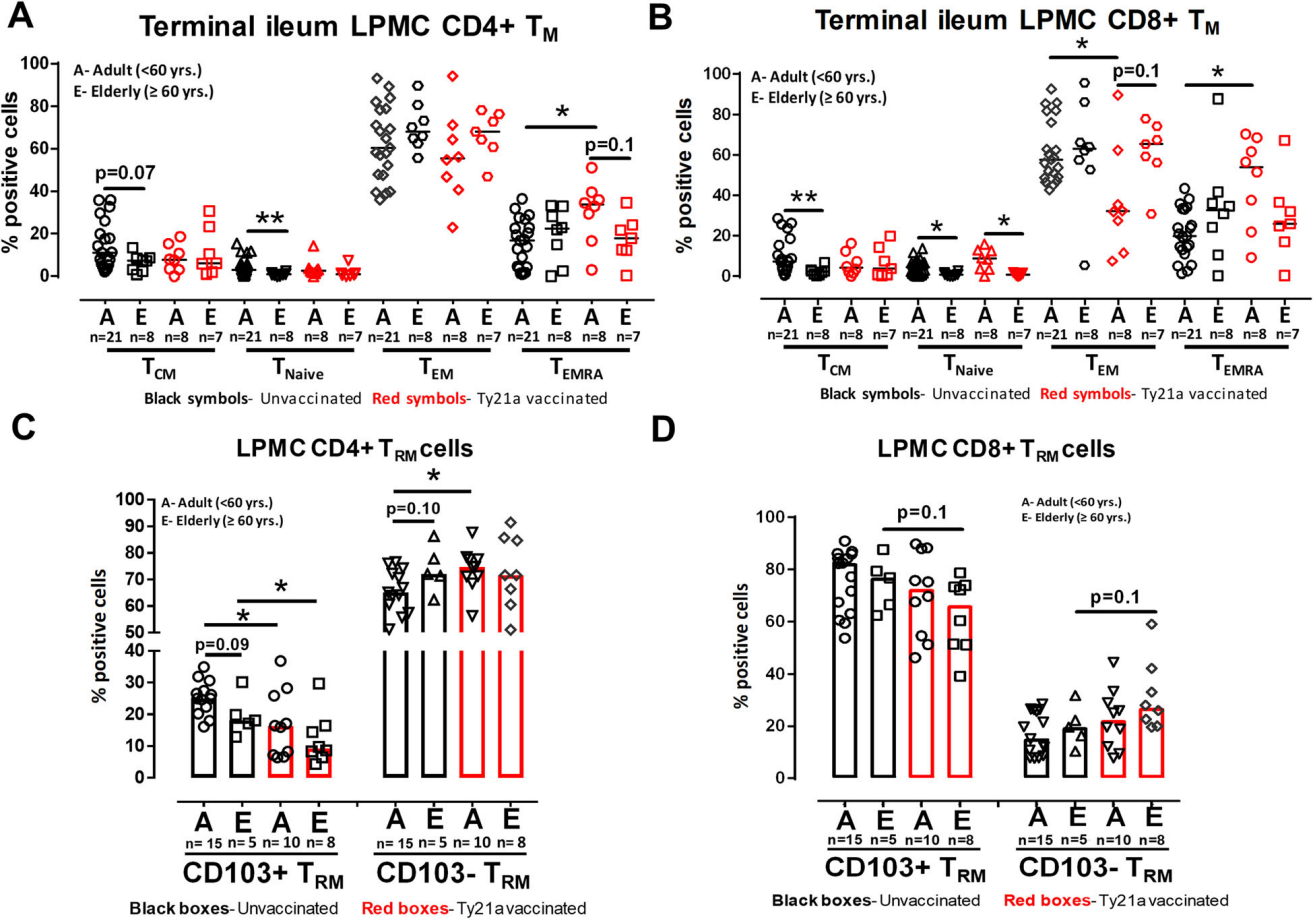
Age differences in frequencies of LPMC memory T cells isolated from Ty21a-vaccinated and unvaccinated volunteers. Comparison of T_M_ subsets including T_CM_ (CD62L+ CD45RA^−^), T_EM_ (CD62L- CD45RA-), T_EMRA_ (CD62L- CD45RA+), and T_naive_ (CD62L+ CD45RA+) between adults (A; < 60 yrs.) and elderly (E; ≥60 yrs.) in TI-LPMC (**a**) CD4+ T and (**b**) CD8+ T cells obtained from Ty21a-vaccinated (red symbols) and unvaccinated (black symbols) volunteers. Frequencies of (**c**) TI-LPMC CD4+ CD103+ and CD4+ CD103- T_RM_ and (**d**) TI-LPMC CD8+ CD69+ CD103+ (CD8+ T_RM_) and CD8+ CD69+ CD103- T cells were determined and compared between adults and elderly volunteers following Ty21a immunization. Median values for each group are denoted as horizontal black bars. Significant differences are denoted with *P < 0.05, **P < 0.005. Trends to exhibit significance values are indicated by their *p*-values

Subsequently, we determined the frequencies of LPMC memory T (T_M_) subsets namely T-central/memory (T_CM_) (CD62L+ CD45RA-), T-effector/memory (T_EM_) (CD62L- CD45RA-), T_EM_-CD45RA+ (T_EMRA_) (CD62L- CD45RA+) and T naïve (T_Naïve_) (CD62L+ CD45RA+) using CD45RA and CD62L markers to define these subsets in elderly and adult volunteers. We observed that in unvaccinated volunteers, LPMC CD4+ T_CM_ (trend; *p* = 0.07) and T_Naive_ (significant; *p* < 0.05) subsets exhibited lower frequencies in the elderly as compared to adults (Fig. 1a). However, no statistically significant differences were observed between LPMC CD4+ T_EM_ and T_EMRA_ in unvaccinated volunteers (Fig. 1a). Following Ty21a immunization, no statistically significant differences were observed in LPMC CD4+ T_M_ subsets except for a trend (*p* = 0.1) to show decreases in the frequencies of CD4+ T_EMRA_ (Fig. 1a). Similar observations for CD8+ T_M_ subsets in unvaccinated volunteers were noted with LPMC CD8+ T_CM_ and T_Naive_ frequencies which were significantly (*p* < 0.05) lower in the elderly (Fig. 1b). However, following Ty21a immunization, no statistically significant differences were observed within LPMC CD4+ T_CM_ and T_EMRA_ subsets between the two groups (Fig. 1a). Interestingly, following Ty21a immunization, LPMC CD8+ T_Naive_ showed statistically significant (*p* < 0.05) lower levels in the elderly as compared to adult volunteers while CD8+ T_EM_ showed a trend (*p* = 0.1) to exhibit increases in the elderly compared to adult volunteers following Ty21a immunization (Fig. 1b).

We next focused on the newly defined tissue resident memory T cells using CD69 and CD103 markers to delineate both CD4+ and CD8+ T_RM_ subsets. Interestingly, in unvaccinated volunteers, we noted that there was a trend (*p* = 0.09) for lower frequencies of CD103+ CD4+ T_RM_ in the elderly as compared to adults while the frequencies of CD103- CD4+ T_RM_ showed a trend (*p* = 0.1) to be higher in the elderly as compared to adults (Fig. 1c). Following Ty21a immunization, no statistically significant differences were observed in the frequencies of CD103+ and CD103- CD4+ T_RM_ between adults and the elderly. The frequencies of both LPMC CD8+ T_RM_ and CD103- CD8+ T cells were not significantly different between elderly and adult volunteers regardless of Ty21a vaccination (Fig. 1d). Of note, following Ty21a immunization, we observed significant (*p* < 0.05) decreases in the frequencies of CD103+ CD4+ T_RM_ in both adults and the elderly and a significant (*p* < 0.05) increase in the frequency of CD103- CD4+ T_RM_ in adults but not in the elderly (Fig. 1c). A similar trend (*p* = 0.1) to show increases in the elderly was observed in CD103- CD8+ T_RM_ (Fig. 1d).

### Age-association of LPMC CD4+ and CD8+ tissue resident cells

**Fig. 2:**
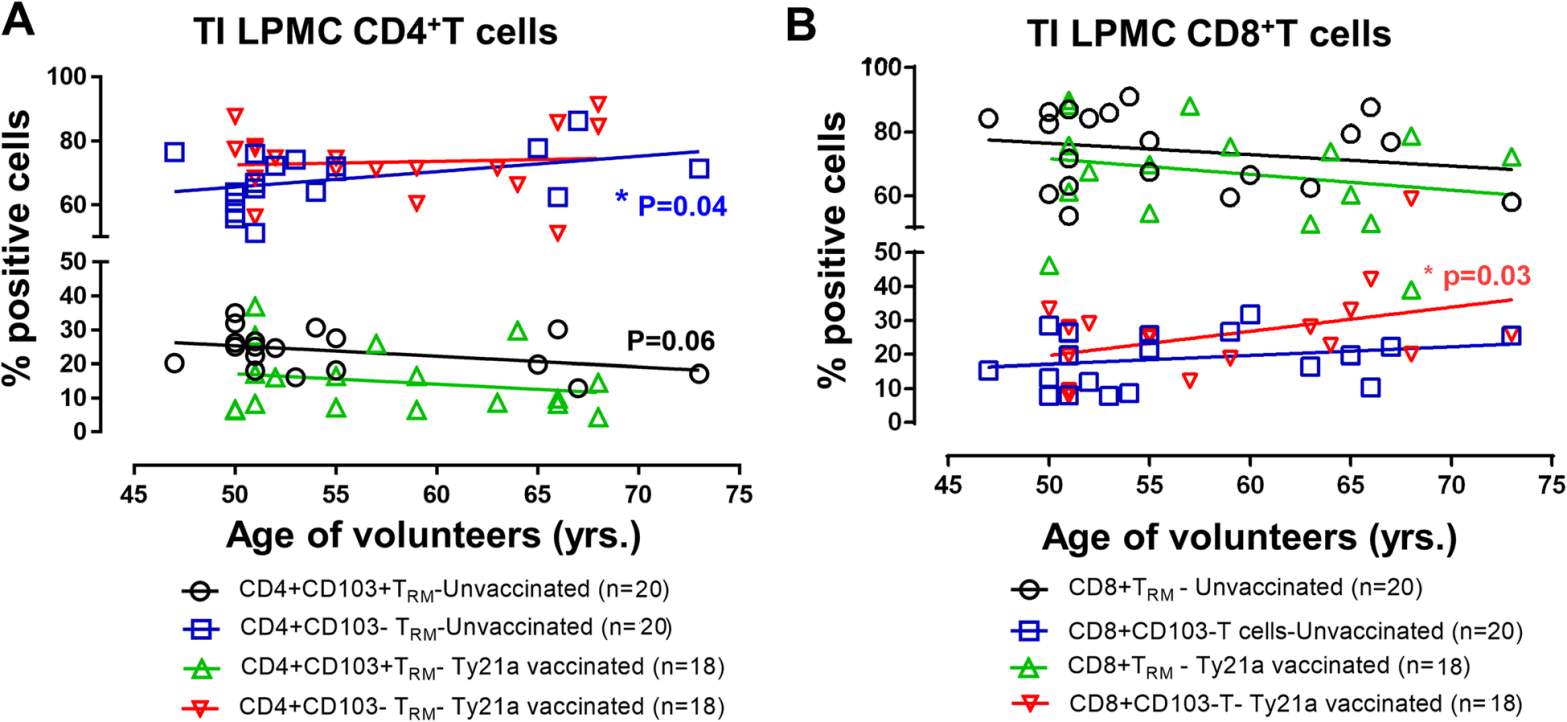
Age-dependent association of CD4+ and CD8+ T_RM_ frequencies and T cell subsets in terminal ileum mucosa following oral Ty21a immunization. Correlations between age and the frequencies of tissue resident T cells were determined using Pearson’s analysis. **a** Unvaccinated LPMC CD4+ CD103+ T_RM_ (black symbols), and CD4+ CD103- T_RM_ (blue symbols), as well as Ty21a-vaccinated LPMC CD4+ CD103+ T_RM_ (green symbols) and CD4+ CD103- T_RM_ (red symbols) frequencies were correlated to the age of the volunteers. **b** Similarly, unvaccinated LPMC CD8+ T_RM_ cells (black symbols), and CD8+ CD103- T cells (blue symbols), and Ty21a-vaccinated LPMC CD8+ T_RM_ (green symbols) and CD8+ CD103- T cells (red symbols) frequencies were correlated to the age of the volunteers. Significant correlations are denoted with * *P* < 0.05. Trends to exhibit significant correlations are indicated by their *p*-values

Tissue resident memory cells plays an important role in protective immunity against pathogens. However, it is unknown whether terminal ileum T_RM_ frequencies are correlated with age in unvaccinated and Ty21a-vaccinated individuals. Here we used Pearson's correlation analysis to determine the degree of association between age and T_RM_ frequencies in Ty21a-vaccinated and unvaccinated individuals. Interestingly, in unvaccinated volunteers, we observed that LPMC CD103- CD4+ T_RM_ displayed a significant (*p* < 0.05) positive correlation with age of volunteers while CD103+ CD4+ T_RM_ displayed a trend (*p* = 0.06) towards a negative correlation with increasing age (Fig. 2a). No significant associations were noted between age and these two cell subsets frequencies following Ty21a immunization (Fig. 2a). Next, we determined associations between age and CD8+ T_RM_ frequencies. No significant associations were noted between age and frequencies of CD8+ T_RM_ and CD103- CD8+ T cells in unvaccinated volunteers (Fig. 2b). However, following Ty21a immunization, we observed a significant (*p* = 0.03) positive correlation between the frequencies of CD103- CD8+ T cells and age (Fig. 2b).

**Fig. 3:**
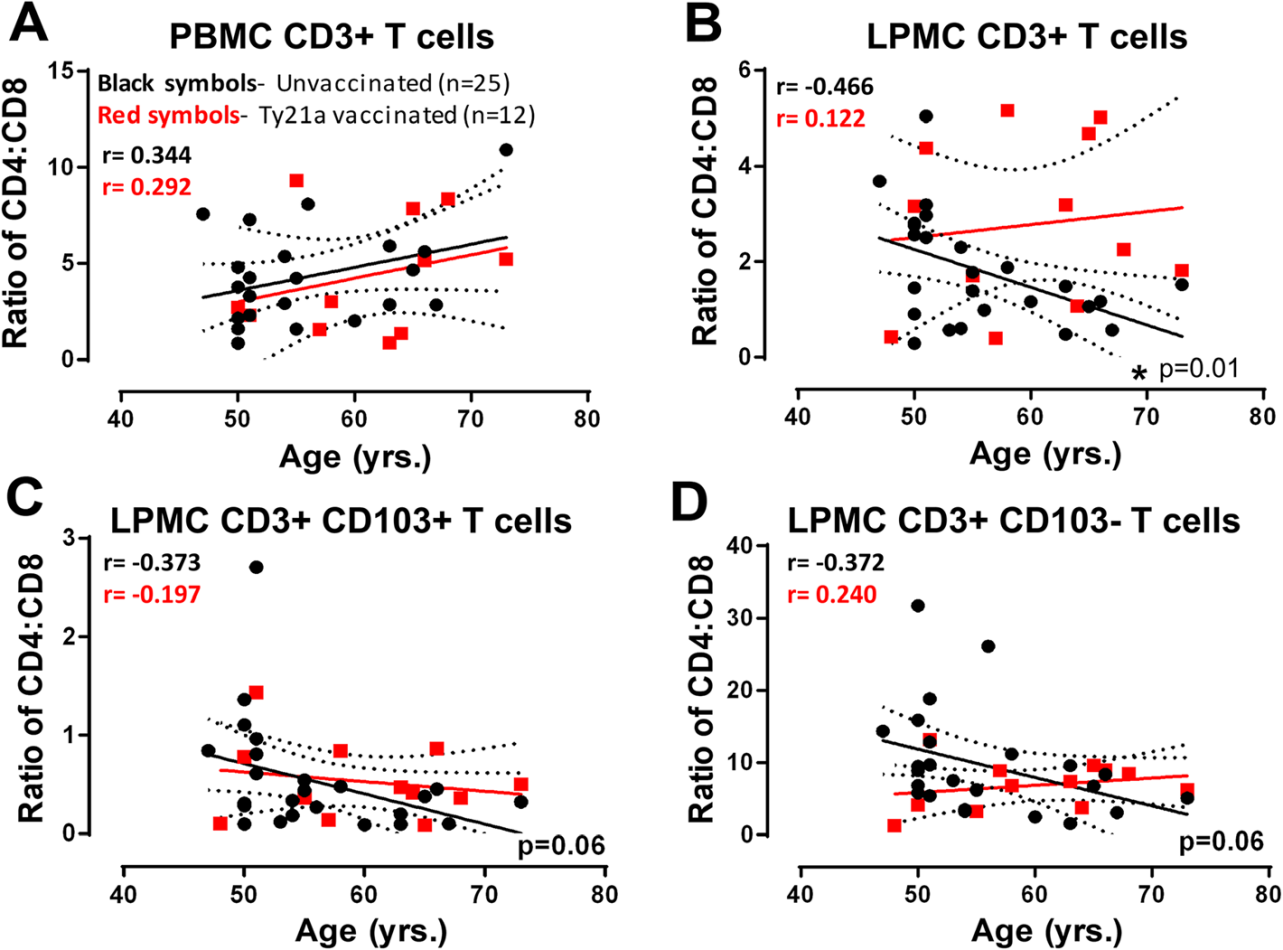
Age-dependent correlation of the ratios of CD4 to CD8 cells among CD3+ T cells in peripheral blood and in CD103+ and CD103- subsets in LPMC isolated from TI biopsies. The ratio of CD4 to CD8 cells were correlated to age in (**a**) PBMC CD3+ T cells, (**b**) LPMC CD3+ T cells, (**c**) LPMC CD3+ CD103+ T cells and (**d**) LPMC CD3+ CD103- T cells. Significant correlations and trends to show correlations are indicated with * and/or their p-value, respectively

Multiple studies have examined CD4:CD8 ratios in various populations including France, Austria, Spain, USA and China and have shown either increases, decreases or no changes depending on the populations evaluated [31–34]. It is widely accepted that inverted CD4:CD8 ratios represent immune risk factors related to fewer B cells, expansion of late-differentiated or senescent T cells (CD8+ CD28-), and higher human cytomegalovirus (HCMV) seropositivity [35]. Of note, CD4:CD8 ratios have been usually determined in peripheral blood rather than in tissues and never following oral Ty21a immunization. Thus, we determined the CD4/CD8 ratios in both blood and terminal ileum LPMC total CD3+ T cells, as well as in CD103+ and CD103- T cell subsets in both unvaccinated and vaccinated volunteers. Similar to the data reported by Lin et al., 2016 [34], we observed that the ratio of CD4/CD8 in PBMC appears to increase with age in our Baltimore cohort; however, these changes did not reach statistical significance. However, no differences were observed between unvaccinated and vaccinated groups (Fig. 3a). In contrast, in TI LPMC CD3+ T cells obtained from unvaccinated volunteers, the CD4/CD8 ratios decrease with age as shown by a significantly (*p* = 0.01) negative correlation (*r* = − 0.466) (Fig. 3b). Of note, Ty21a immunization eliminated this negative correlation, suggesting that immunization elicited an influx of CD3+ CD4+ cells to the TI, particularly in elderly individuals (Fig. 3b). We next determined whether the effects of aging on CD4/CD8 ratios in TI LPMC were primarily the results of effects on CD103+ and/or CD103- T cells (resident T cells). We observed that in unvaccinated individuals, both CD103+ and CD103- CD3+ cells exhibited a trend (*p* = 0.06) to show negative correlations with increasing age (Fig. 3c, d). However, as noted for total CD3+ cells, oral Ty21a immunization eliminated these trends in both subsets (Fig. 3c, d). Thus, oral Ty21a immunization appears to influence the reservoir of local T cells in the elderly as well as in adults.

### Age-differences in single-expressing and multifunctional LPMC CD4+ and CD8+ T_EM_*S*. Typhi-specific responses following Ty21a immunization

**Fig. 4:**
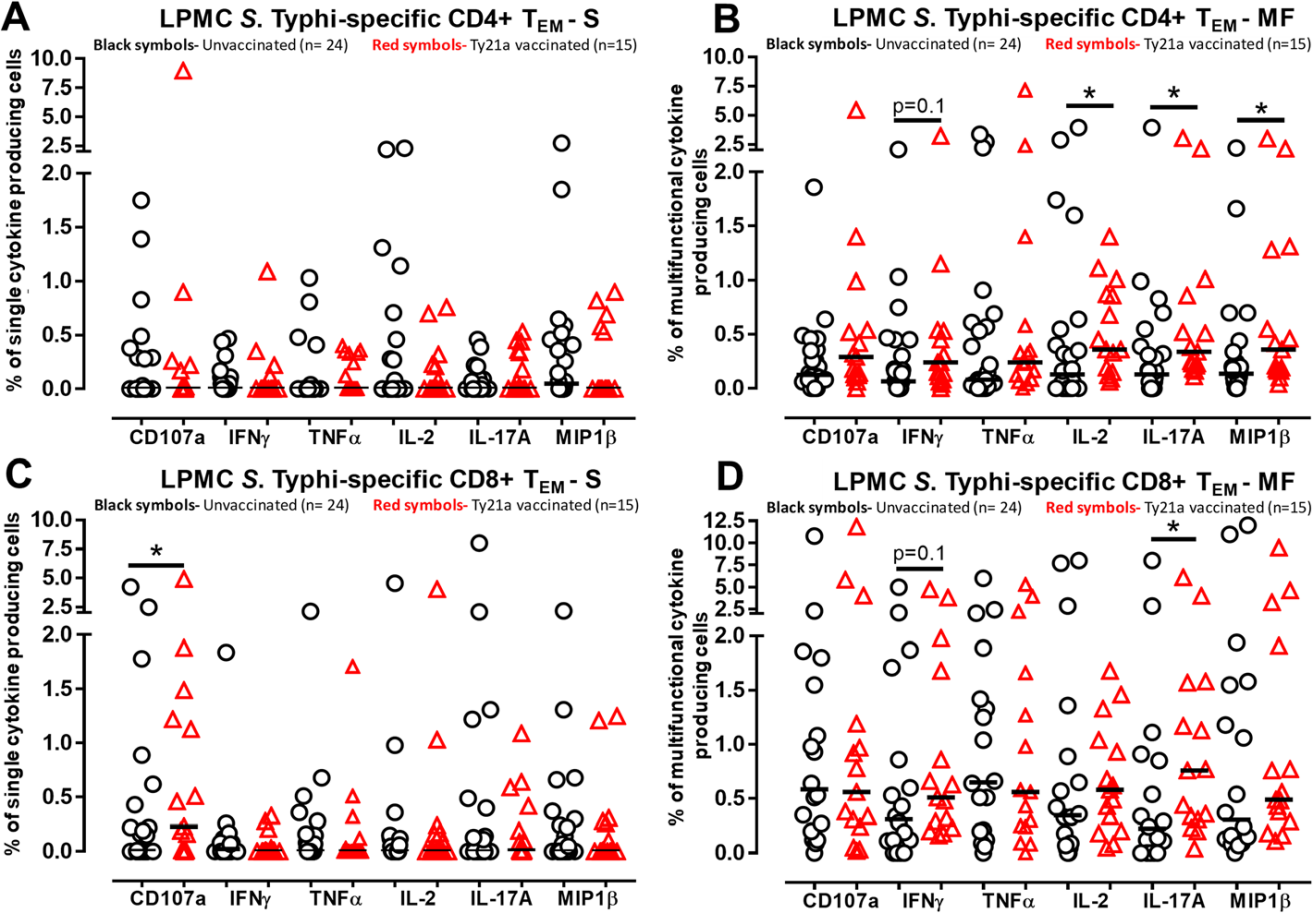
Multifunctional (MF) and single-expressing (S) *S.* Typhi-specific CD4+ and CD8+ T_EM_ responses following Ty21a immunization. Net *S.* Typhi-specific CD4^+^ and CD8+ T_EM_ responses were calculated using the FCOM function of Winlist and stratified into S and MF responses. Comparison of TI LPMC *S.* Typhi-specific (**a**) CD4+ T_EM_ S, (**b**) CD4+ T_EM_ MF, (**c**) CD8+ T_EM_ S and (**d**) CD8+ T_EM_ MF responses between Ty21a-vaccinated (red symbols) and unvaccinated (black symbols) volunteers were determined with significant differences shown (*P < 0.05). Trends to exhibit significance values are indicated by their p-values. Horizontal black bars represent median values

Using the Ty21a human vaccination model, we have previously reported the induction of *S.* Typhi-specific responses in TI LPMC CD4+ [25] and CD8+ [36] T_M_ subsets. However, age differences were not addressed in these studies. Because we observed altered T_M_ frequencies in elderly LPMC (Figs. 1, 2 and 3), we hypothesized that LPMC *S*. Typhi-specific responses would also be affected in the elderly compared to adult volunteers following Ty21a immunization. To test that hypothesis, we stratified and analyzed LPMC *S*. Typhi-specific T_M_ subsets responses in adults (< 60 yrs.) and in the elderly (≥60 yrs.). Induction of antigen-specific multifunctional T cells have been shown to be associated with favorable disease outcome, higher effector function and higher protective efficacy after immunization compared to monofunctional T cells in various diseases including typhoid fever [37–41]. In addition, multifunctional and monofunctional T cells have been recently shown to exhibit molecular differences [42]. We have shown previously that many significant changes observed following Ty21a immunization occur, depending on the *S.* Typhi-specific responses being evaluated, in single-expressing (S) and/or multifunctional (MF) LPMC CD4+ and CD8+ T_EM_ cells [25–28, 36]. Thus, we examined the S and MF LPMC CD4+ and CD8+ T_EM_ S. Typhi-specific responses in this cohort. To this end we used the Winlist FCOM function to simultaneously analyze production of multiple cytokines/chemokines (i.e., IFNγ, TNFα, IL-2, IL-17A, and MIP1β) and expression of CD107a (a marker of cytotoxicity) in individual *S*. Typhi-specific responding cells and classified them as either single cytokine producers/CD107a expressors (S) or multifunctional (sum of double, triple, quadruple, quintuple or sextuple cytokine producers/CD107a expressors) (MF). No statistically significant differences were observed in CD4 + T_EM_ -S for any of the responses evaluated following Ty21a immunization (Fig. 4a). However, we observed that LPMC CD4+ T_EM_-MF elicited higher levels of cytokines (IFNγ- *p* = 0.1; IL-2 -*p* < 0.05; IL-17A- *p* < 0.05; and MIP1β- *p* < 0.05) in Ty21a-vaccinated than in unvaccinated volunteers (Fig. 4b). For LPMC CD8+ T_EM_, we observed that Ty21a induced significant (*p* < 0.05) higher level of CD107a-S, IL-17A-MF and a trend (*p* = 0.1) for higher levels of IFNγ-MF in vaccinated than unvaccinated volunteers (Fig. 4c-d). Of note, these findings in the present cohort confirmed our previous reports in which we studied populations which included both adults (< 60 yrs.) and elderly (≥60 yrs.) volunteers.

**Fig. 5:**
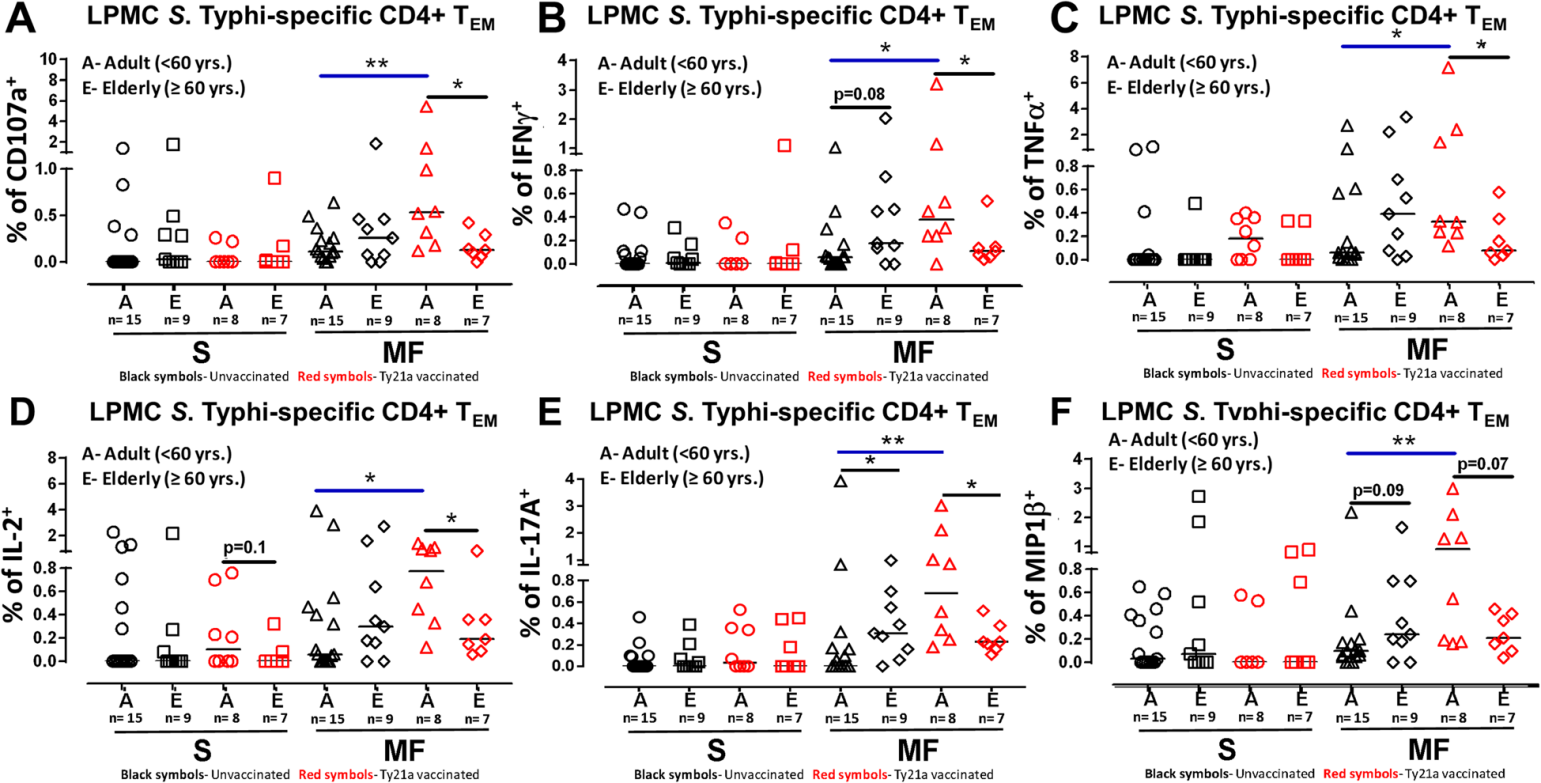
Multifunctional (MF) and single-expressing (S) *S.* Typhi-specific CD4+ T_EM_ responses in adults compared to elderly volunteers following Ty21a immunization. Net *S.* Typhi-specific CD4^+^ T_EM_ responses were calculated using the FCOM function of Winlist and stratified into single-expressing (S) and MF responses. Comparison of TI LPMC *S.* Typhi-specific CD4+ T_EM_ responses between adults (A- < 60 yrs.) and elderly (E- ≥ 60 yrs.) volunteers in (**a**) CD107a+, (**b**) INF-γ+, (**c**) TNF-α+, (**d**) IL-17A+, (**e**) IL-2+, and (**f**) MIP-1β + S and MF responses in Ty21a-vaccinated (red symbols) and unvaccinated (black symbols) volunteers with significant differences shown (*P < 0.05). Trends to exhibit significance values are indicated by their p-values. Horizontal black bars represent median values

We next examined the effect of age on the characteristics and levels *S.* Typhi-specific S and MF LPMC CD4+ and CD8+ T_EM_ following Ty21a vaccination by dividing the present cohort into elderly (≥60 yrs.) and adult (< 60 yrs.) groups. First, we analyzed the net *S*. Typhi-specific responses of LPMC CD4 + T_EM_ between the elderly and adult groups. Interestingly, we observed statistically significant (*p* < 0.05) lower levels of MF IFNγ, TNFα, IL-2, IL-17A and expression of CD107a in the elderly than in adults following Ty21a immunization (Fig. 5a-e). In addition, we observed a trend (*p* = 0.07) to show lower responses in CD4+ T_EM_ MIP1β + in the elderly compared to adult volunteers following Ty21a immunization (Fig. 5f). No statistically significant differences or trends were observed in CD4+ T_EM_-S for CD107a, IFNγ, TNFα, IL-17A and MIP1β (Fig. 5a-c, e, f). A trend (*p* = 0.1) to show decreased responses for IL-2-S was also observed in elderly as compared to adult volunteers following Ty21a immunization (Fig. 5d). Interestingly, we also observed that in unvaccinated volunteers, the levels of CD4+ T_EM_ MF were higher (IFNγ (*p* = 0.08); IL-17A (p < 0.05) and MIP1β (*p* = 0.09) in the elderly than in adults (Fig. 5b, e-f). Thus, we conclude that aging influences the elicited LPMC *S*. Typhi specific CD4+ T responses following Ty21a immunization.

**Fig. 6:**
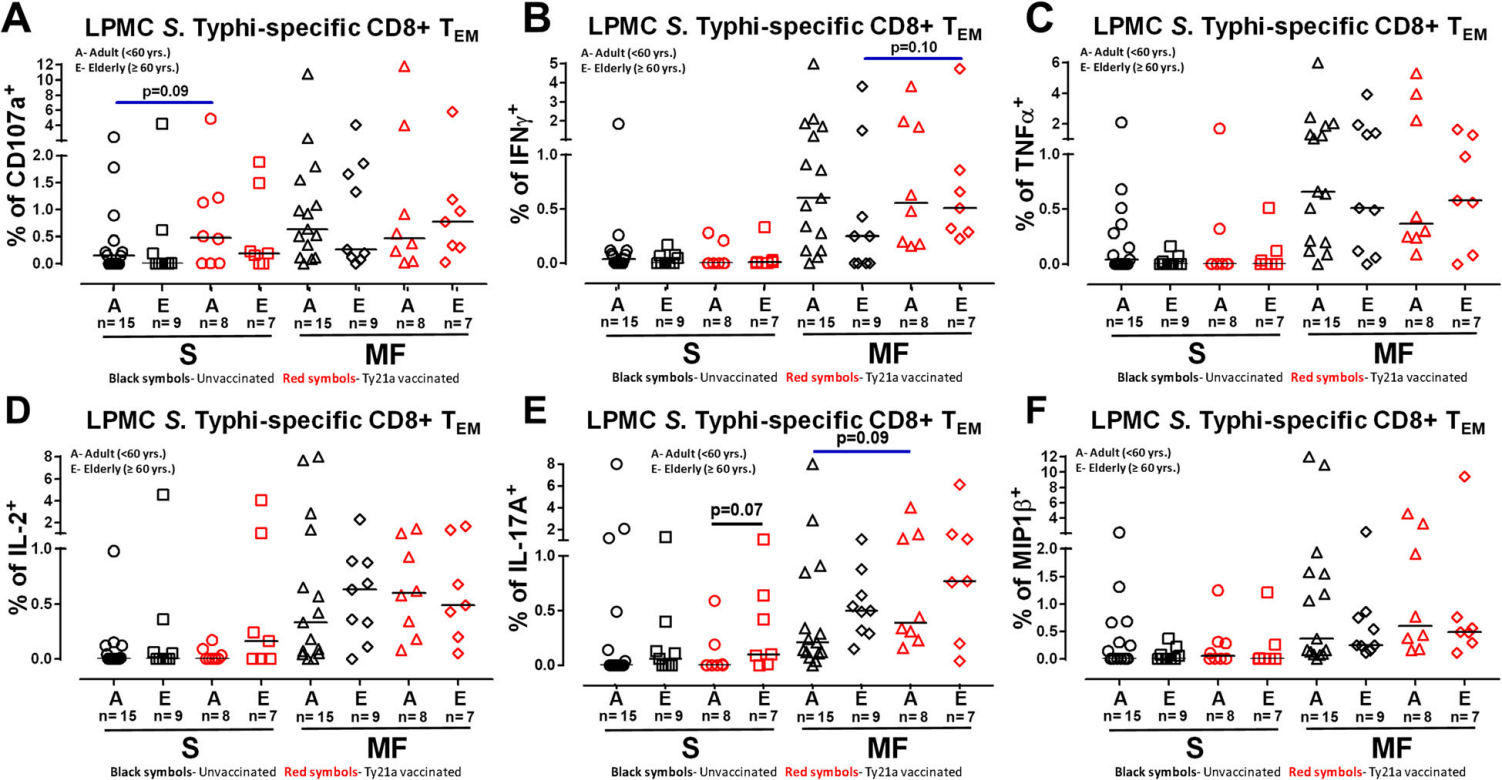
LPMC multifunctional (MF) and single-expressing (S) *S.* Typhi–specific CD8+ T_EM_ responses in adults compared to elderly volunteers following Ty21a immunization. Net *S.* Typhi-specific CD8+ T_EM_ responses were calculated using the FCOM function of Winlist and stratified into S and MF responses. Comparison of TI LPMC *S.* Typhi-specific CD8+ T_EM_ responses in (**a**) CD107a+, (**b**) INF-γ, (**c**) TNF-α+, (**d**) IL-17A+, (**e**) IL-2+, and (**f**) MIP-1β + S and MF between adults (A- < 60 yrs.) and elderly (E- ≥ 60 yrs.) volunteers that were either orally vaccinated with Ty21a (red symbols) or unvaccinated (black symbols). Trends to exhibit significant values are indicated by their p-values. Horizontal black bars represent median values

In contrast, analysis of net *S*. Typhi-specific responses by LPMC CD8+ T_EM_ demonstrated no statistically significant differences in the levels of MF IFNγ, TNFα, IL-2, IL-17A, MIP1β or expression of CD107a between the elderly and adult individuals following Ty21a immunization (Fig. 6a-f). No statistically significant differences in CD8+ T_EM_-S were observed for IFNγ, TNFα, IL-2, MIP1β and expression of CD107a (Fig. 6a-d, f) except for a trend (*p* = 0.07) in IL-17A-S to exhibit higher levels in the elderly as compared to adults (Fig. 6e). In addition, we observed trends to show increases in *S*. Typhi specific CD8+ T_EM_ (IL-17A-MF (*p* = 0.09) and CD107a-S (*p* = 0.09)) in Ty21a vaccinated than in unvaccinated volunteers in adults but not in elderly volunteers (Fig. 6a-b, e). In addition, a trend (*p* = 0.1) in IFNγ-MF to exhibit higher levels in Ty21a vaccinated than unvaccinated elderly volunteers was detected (Fig. 6b).

### Age differences in *S*. Typhi-specific responses by LPMC-CD4+ T_RM_ subsets following Ty21a immunization

**Fig. 7:**
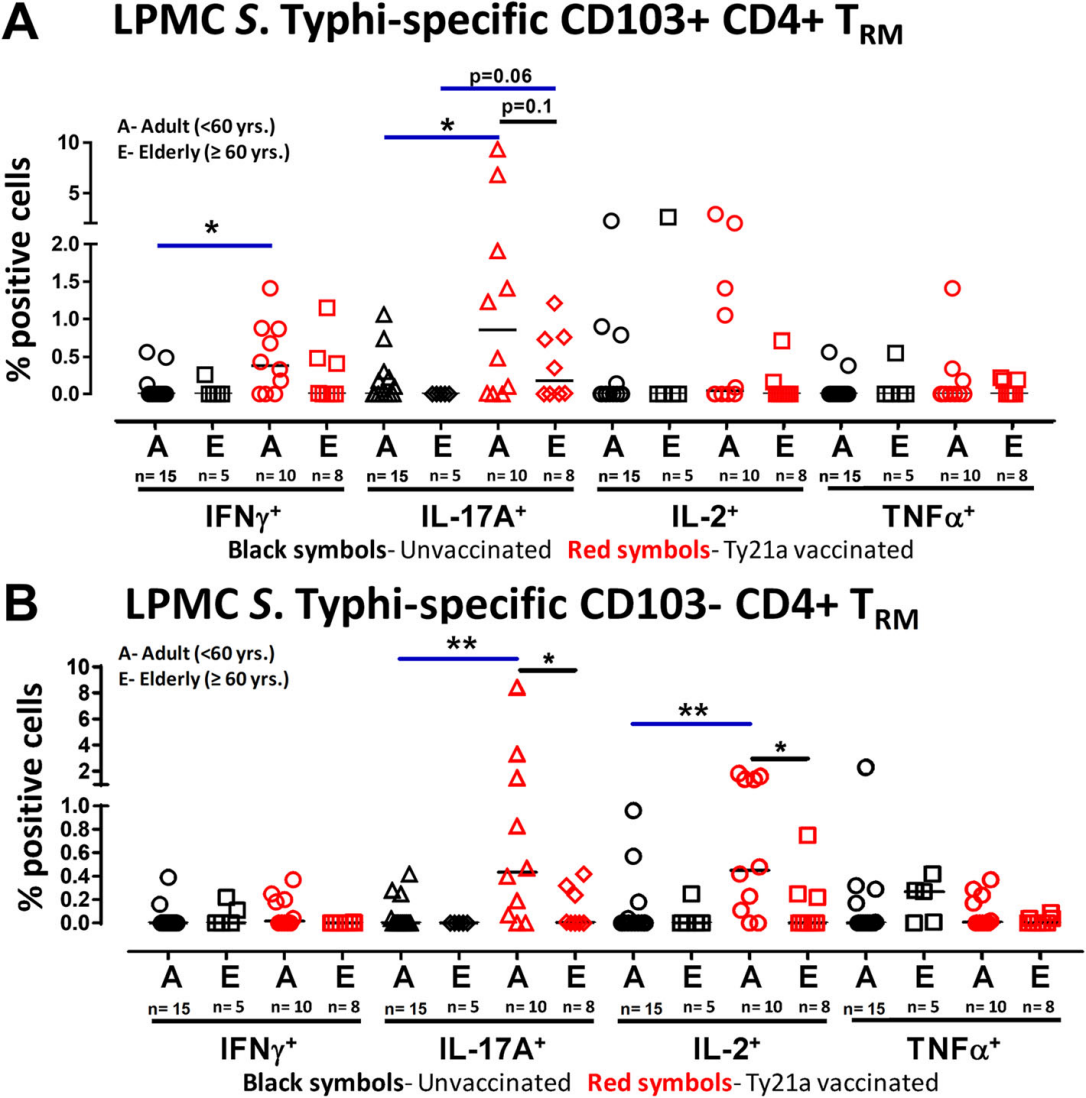
*S*. Typhi-specific responses of TI LPMC CD69+ CD103+ CD4+ T_RM_ and CD69+ CD103- CD4+ T_RM_ subsets in healthy adults and elderly volunteers following oral Ty21a-immunization. The net percentages of *S*. Typhi-specific responses (IFNγ+, IL-17A+, IL-2+, and TNFα+) in (**a**) CD69+ CD103+ CD4+ T_RM_ and (**b**) CD69+ CD103- CD4+ T_RM_ subsets were compared between adults (A- < 60 yrs.) and elderly (E- ≥ 60 yrs.) volunteers who were either Ty21a-vaccinated (red symbols) or unvaccinated (black symbols) with significant differences (*P < 0.05) indicated. Trends to exhibit significance are indicated by their p-values. Horizontal bars (black) represent median values

Tissue resident memory T cells contribute to local immune responses at the site of infection following infection and ultimately to protective immunity against pathogens [43, 44]. The composition and functions of CD4+ T cells is one of the major changes observed during aging [4, 45]. For example, classical CD4+ T cell subsets has been shown to be affected during the aging process by reducing their proliferation and production of IL-2 [46]. In addition, there is an increase in regulatory T cells (T_regs_) with age that contribute to decrease responsiveness of effector T cells [47]. However, not much is known about the impact of aging on LPMC T_RM_ particularly in the context of oral immunization. Here we took advantage of the human Ty21a immunization model to determine the effect of aging on human T_RM_ antigen-specific responses. We first determined the effect of aging on the responses of LPMC CD4+ T_RM_ subsets by examining net *S*. Typhi-specific responses in elderly and adult volunteers. As previously shown, using CD69 and CD103 markers, TI-LPMC CD4+ T_RM_ are comprised of two populations, namely CD103- CD69+ (~ 70%) and CD103+ CD69+ (~ 20%) (Fig. S1A). We observed that CD103+ CD4+ T_RM_ showed a trend (*p* = 0.1) to exhibit decreased IL-17A responses in elderly than in adult volunteers following Ty21a immunization (Fig. 7a). In contrast, CD103- CD4+ T_RM_ displayed significant (*p* < 0.05) decreases in the levels of IL-17A and IL-2 in the elderly compared to adults following Ty21a immunization (Fig. 7b). Of importance, significantly increased responses in adults were observed for IFN-γ (*p* < 0.05) and IL-17A (p < 0.05) in CD4+ CD103+ CD69+ T_RM_ and for IL-17A (*p* < 0.05) and IL-2 (*p* < 0.005) in CD4+ CD103- CD69+ T_RM_ following Ty21a immunization (Fig. 7a-b).

To further investigate the differences in LPMC CD103+ and CD103- CD4+ T_RM_ *S*. Typhi-specific responses between the elderly and adults, we used the Winlist FCOM function to analyze multiple cytokines (i.e., IFNγ, IL-17A, IL-2, and TNFα) in individual *S*. Typhi-specific responding cells and classified them as either single cytokine producer (S) or multifunctional (Sum of double, triple, and quadruple cytokine producers) (MF). First, we analyzed the net *S*. Typhi-specific LPMC CD103+ CD4+ T_RM_ MF responses and found no significant differences in IFN-γ, IL-17A and TNFα MF *S*. Typhi-specific responses between elderly and adult volunteers following Ty21a immunization (Table S1). However, we noted a trend (*p* = 0.1) to exhibit decreases in the production of IL-2-MF obtained from LPMC CD103+ CD4+ T_RM_ in the elderly compared to adults following Ty21a immunization (Table S1). In contrast, *S*. Typhi-specific LPMC CD103+ CD4+ T_RM_ S responses displayed a trend (*p* = 0.1) to show increases in IFNγ-S but decreases (p = 0.1) in IL-17A-S in the elderly compared to adult Ty21a-vaccinated volunteers (Table S1). No significant differences in IL-2-S and TNFα-S production from LPMC CD103+ CD4+ T_RM_ were noted between elderly and adults following Ty21a immunization (Table S1).

Next, we analyzed the net *S*. Typhi-specific LPMC CD103- CD4+ T_RM_ MF responses and found that no significant differences in IFN-γ and TNFα MF *S*. Typhi-specific responses between elderly and adults following Ty21a immunization (Table S1). However, we noted a trend (*p* = 0.1) to show decreases in the production of IL-17A-MF and IL-2-MF, as well as IL-17A-S in CD103- CD4+ T_RM_ in the elderly compared to adult volunteers following Ty21a immunization (Table S1). No statistically significant differences in *S*. Typhi-specific IFNγ-S, IL-2-S and TNFα-S production from LPMC CD103- CD4 + T_RM_ were observed among elderly and adult volunteers following Ty21a immunization (Table S1).

Interestingly, we noted that both CD4+ T_RM_ subsets, particularly CD103- CD4+ T_RM_, exhibited some differences in the α-CD3/CD28 stimulation responses between elderly and adult volunteers. For example, CD4 + T_RM_ subsets responses examined in both age groups showed that α-CD3/CD28 beads stimulated equally well both adult and elderly CD103+ CD4 + T_RM_ to produce high multifunctional IFNγ, IL-17A, IL-2 and TNFα subsets in the two age groups (Table S1). Oral Ty21a-immunization did not significantly influence any of the measured cytokine responses (Table S1). Interestingly, both adult and elderly CD103- CD4+ T_RM_ were stimulated equally well by α-CD3/CD28 beads to produce multifunctional IFNγ, IL-17A+, IL-2+ and TNFα MF responses (Table S1). However, CD103- CD4+ T_RM_ showed significantly (*p* < 0.05) lower levels of IFNγ-S production, and a trend (*p* = 0.1) to exhibit lower levels of IL-17A following α-CD3/CD28 beads stimulation in elderly than in adult Ty21a-vaccinated volunteers (Table S1). No statistically significant differences between adult and elderly volunteers were observed following stimulation of CD103- CD4+ T_RM_ by α-CD3/CD28 beads in the production of IL-2 and TNFα (Table S1). These granular data suggest that aging and oral Ty21a-immunization have the potential to somewhat influence intrinsic differences of LPMC CD4+ T_RM_ resulting in distinct stimulatory characteristics.

### Age differences in *S*. Typhi-specific responses by LPMC CD8+ T_RM_ subsets following Ty21a immunization

**Fig. 8:**
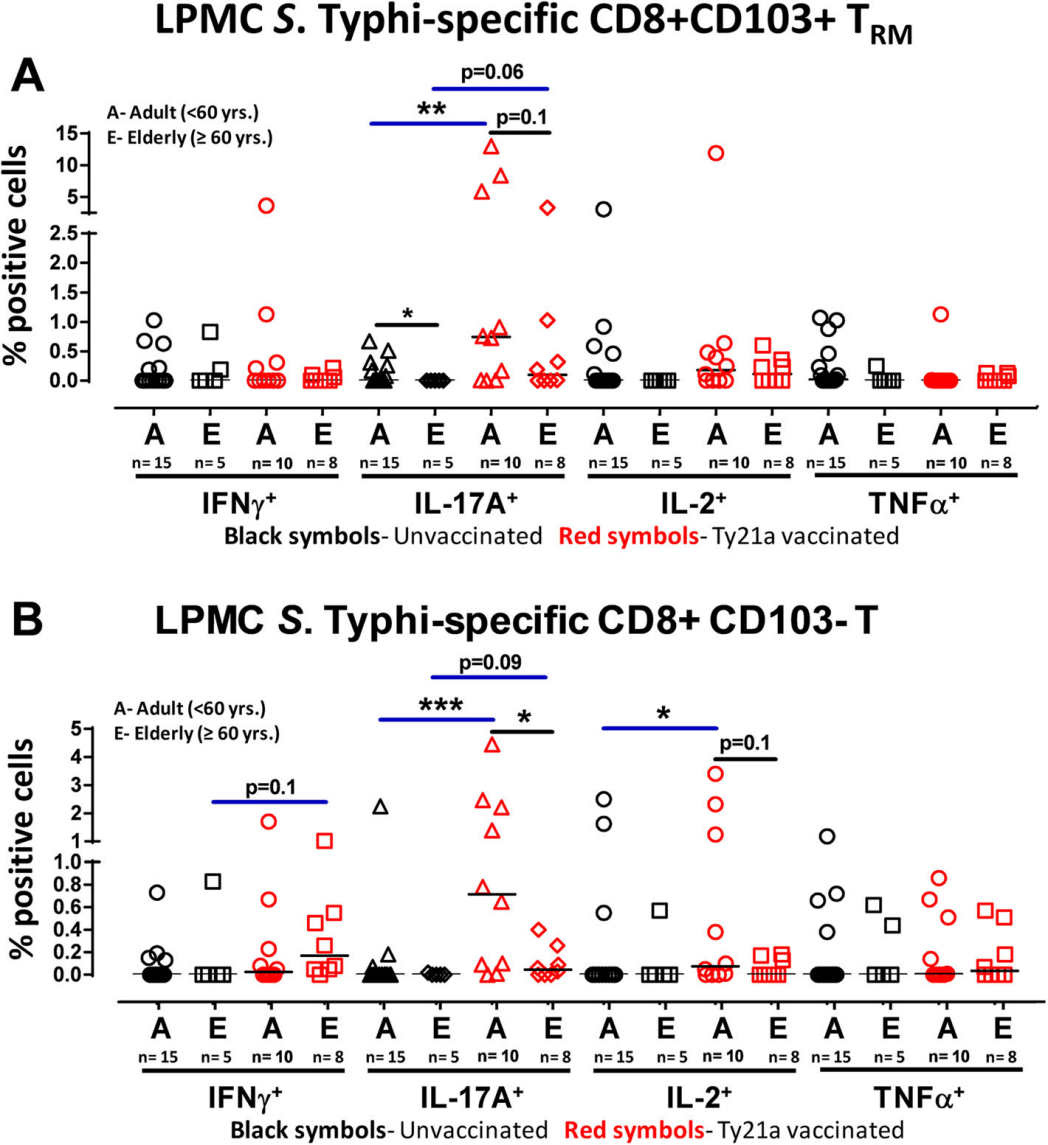
*S*. Typhi-specific responses of terminal ileum LPMC CD8+ T_RM_ and CD8+ CD69+ CD103- T cell subsets in healthy adults and elderly volunteers following oral Ty21a immunization. The net percentages of *S*. Typhi-specific responses (IFNγ+, IL-17A+, IL-2+, and TNFα+) in (**a**) CD8+ T_RM_ and (**b**) CD8+ CD69+ CD103- T cell subsets were compared between adults (A- < 60 yrs) and elderly (E- ≥ 60 yrs.) volunteers who were either Ty21a-vaccinated (red symbols) or unvaccinated (black symbols). Significant differences (*P < 0.05; **P < 0.005; ***P < 0.0005) are indicated. Trends to exhibit significance are indicated by their *p*-values. Horizontal bars (black) represent median values

In humans, the change in proportions of naïve and memory CD8+ T cells is a prominent feature of the aging process. In addition, CD8+ T cells among PBMC have been shown to have impaired cell proliferation, higher levels of CD57 expression (a senescence marker) and decreased cell activation markers [48]. CD8+ T_RM_ is one of the major T cell subsets located in the terminal ileum (main site of infection for *S*. Typhi). However, no information is available regarding its role and contribution in the *S*. Typhi-specific responses elicited in the elderly following oral Ty21a immunization. Since we did not observe significant changes in the frequencies of LPMC CD8+ T_RM_ and CD103- CD8+ T cells between adult and elderly, we hypothesized that both CD8+ T_RM_ and CD103- CD8+ T cells would respond similarly in elderly and adults following Ty21a immunization. To test this hypothesis, we compared *S*. Typhi-specific responses of CD8+ T_RM_ and CD103- CD8+ T cells between elderly and adult volunteers following Ty21a immunization. We observed that CD8+ T_RM_ showed a trend (*p* = 0.1) to exhibit decreases in IL-17A, but not IFNγ, IL-2 and TNFα production in elderly as compared to adult volunteers following Ty21a immunization (Fig. 8a). In contrast, we observed that CD103- CD8+ T cells displayed significantly (*p* < 0.05) lower levels of IL-17A, and a trend (p = 0.1) towards lower IL-2 responses in elderly than adult volunteers following Ty21a immunization (Fig. 8b).

To further investigate the differences in LPMC CD8+ T_RM_ and CD103- CD69+ CD8+ T cells *S*. Typhi-specific responses between elderly and adult volunteers, we analyzed the data using Winlist FCOM function as described above for CD4+ T_RM_ cells. First, we analyzed the net *S*. Typhi-specific LPMC CD8+ T_RM_ MF responses and found no statistically significant differences in IFN-γ, IL-17A, IL-2 and TNFα MF responses between elderly and adults following Ty21a immunization (Table S2), except for a trend (*p* = 0.1) towards lower levels of IL-2 MF cells in elderly than in adults (Table S2). Additionally, LPMC CD8+ T_RM_ S responses displayed trends (p = 0.1) to show decreased IL-17A and TNFα but not in IFNγ and IL-2, responses in Ty21a-vaccinated elderly compared to adult volunteers (Table S2). Next, we analyzed net *S*. Typhi-specific LPMC CD103- CD8+ T S and MF responses. Significant (*p* < 0.05) decreases in IL-17A-S, but not in IFN-γ, IL-2 and TNFα S and MF responses were observed between elderly and adults following Ty21a immunization (Table S2).

Remarkably, we observed that following α-CD3/CD28 stimulation both CD8+ T_RM_ and CD103- CD8+ T cells exhibited differences in their responses between elderly and adult volunteers. Since CD8+ CD28- T cells tend to accumulate during aging and might be present in the intestinal mucosa, we hypothesized that aging might influence the capacity of CD8+ T_RM_ and CD103- CD8+ T cells to respond to stimulation. To address this hypothesis, we evaluated CD8+ T_RM_ and CD103- CD8+ T cells cytokines producing cells obtained from elderly and adults volunteers following stimulation with α-CD3/CD28 beads.

Interestingly, α-CD3/CD28 beads stimulated equally well CD8+ T_RM_ to produce high levels of IFNγ, IL-17A, IL-2 and TNFα S and MF in adults and the elderly (Table S2), except for IL-2-S which was produced at significantly (*p* < 0.05) higher levels (Table S2) and showed a trend (*p* = 0.1) to exhibit higher levels of TNFα-S in elderly than in adult unvaccinated volunteers (Table S2). In contrast, following stimulation with α-CD3/CD28 beads, LPMC CD103- CD8+ T cells produced significantly (*p* < 0.05) higher levels of IFNγ MF in elderly than in adult unvaccinated volunteers (Table S2). However, IL-17-S production by CD103- CD8 + T cells showed decreased levels in the elderly (trend in unvaccinated, *p* = 0.1; significant in Ty21a-vaccinated, *p* < 0.05) than in adults following stimulation with α-CD3/CD28 beads (Table S2). In addition, CD103- CD8+ T cells displayed trends (*p* = 0.1) to show decreases in IL-17-MF in elderly as compared to adults in Ty21a-vaccinated volunteers following α-CD3/CD28 beads stimulation (Table S2). No statistically significant differences were observed in IL-2 S and MF production by CD103- CD8+ T (Table S2). Of note, significantly higher TNFα S (*p < 0.05, **p < 0.005) and MF (*p < 0.05) responses were observed in LPMC CD103- CD8+ T cells obtained from elderly volunteers than from adult volunteers regardless of Ty21a vaccination (Table S2).

### HCMV status and tissue resident receptors (CD69 and CD103) expression in elderly and adults

Because of the suggested role of human cytomegalovirus (HCMV) infection on immunosenescence [49, 50], we also determined whether HCMV seropositivity (an indicator of HCMV exposure) had an effect on *S.* Typhi-specific responses in LPMC. As shown in Table S3, the levels of HCMV seropositivity in adults (< 60 yrs.) were similar in all groups. In adults < 60 yrs. we observed that 52% of unvaccinated and 50% in Ty21a vaccinated volunteers were seropositive for HCMV (Table S3). In the elderly (≥60 yrs.) cohort, HCMV seropositivity was 50 and 38% in unvaccinated and Ty21a-vaccinees respectively (Table S3).

To further understand the factors that influence the differences in *S*. Typhi-specific responses between adults and the elderly, we compared the frequencies of LPMC T cells expressing CD69 or CD103 between the two groups of volunteers following Ty21a immunization or in unvaccinated. We observed no differences in the frequencies of LPMC CD4+ T_EM_ expressing either CD69 or CD103 between adult and elderly volunteers regardless of Ty21a vaccination (Fig. S2A). However, for LPMC CD8+ T_EM_, we detected significantly (*p* < 0.05) higher levels of CD69+ CD8+ T_EM_ cells in the elderly than in adult unvaccinated volunteers (Fig. S2B). Following Ty21a vaccination, no differences were observed in CD69+ CD8+ T_EM_ cells between the two populations, but significant (*p* < 0.005) decreases in CD69+ CD8+ T_EM_ cells were observed in elderly volunteers following Ty21a vaccination (Fig. S2B). Regarding CD103 expression, no statistically significant differences were noted between the two groups except for a trend (*p* = 0.1) to show decreases in the frequencies of CD103+ CD8+ T_EM_ following Ty21a vaccination (Fig. S2B). Thus, oral Ty21a immunization influences differentially the frequencies of cells expressing CD69 and CD103 in adults and the elderly depending on the T cell subset.

## Discussion

Age-dependent changes to the immune system have a clear impact on immune cell function, especially regarding reduced vaccine efficacy as shown by poorer vaccine-induced responses in older individuals [8–11]. Vaccine-mediated protective immunity is generally mediated through the induction of appropriate antibody and cellular immune responses (CMI). Most human studies investigating immunosenescence use cells isolated from peripheral blood (PBMC), largely due to easy accessibility. However, most of the immune cells (e.g., T_RM_) reside in tissues and are functionally and phenotypically different from those present in PBMC. T_RM_ play a crucial role in protective immunity following natural infection and their subsequent secondary exposure [51–53]. However, very limited information is available regarding the impact of aging on T_RM_, particularly in the context of oral immunization (e.g., Ty21a) in the human TI. Here, we examined and compared the responses elicited by Ty21a-immunization on TI LPMC T_M_ and T_RM_ subsets isolated from biopsies of elderly and adult volunteers. We showed that aging influences several immune parameters, including (i) the frequencies of CD4+ T_RM_ subsets regardless of Ty21a immunization; (ii) the ratio of CD4/CD8, which was found to be different in TI LPMC than in blood and that decreases with age; remarkably, Ty21a immunization increased LPMC CD4/CD8 ratios; (iii) the frequencies of TI-LPMC CD4+ T_EM_, which were significantly lower for *S*. Typhi-specific multifunctional responses following oral Ty21a immunization; CD8+ T_EM_ responses were less affected than CD4+ T_EM_; (iv) the frequencies of TI-LPMC CD4+ T_RM_ subsets, which were significantly lower than in adults for *S*. Typhi-specific IL-17A and IL-2 production following Ty21a immunization; and (v) the frequencies of TI-LPMC CD8+ T_RM_ and CD103- CD8+ T cells, which exhibited lower proportions of *S.* Typhi-specific IL-17A and IL-2 than adults. Taken together, these results contribute major novel information of the effects of aging on human TI-LPMC T_M_ and T_RM_ responses following oral Ty21a immunization.

In older individuals, alterations to T cell populations have been implicated in the decline of immunity. For example, the frequencies of PBMC naïve T cells are significantly decreased in the elderly compared to younger individuals, likely as a result of thymic involution [54]. In the intestine, the frequency of naïve cells is already very low. Nevertheless, we were able to document that both TI LPMC CD4+ and CD8+ T_naïve_ populations are significantly lower in the elderly than in adults. A similar finding was reported in the recto-sigmoid colon from elderly individuals [55]. These results indicate that alterations in T cell populations occur not only in blood, but also in tissues. Studies in humans have shown an increased number of memory and effector cells during aging in both peripheral blood CD4+ and CD8+ subsets [56]. Here, we observed lower levels of LPMC CD4+ (trend) and CD8+ (significant) T_CM_, but no significant differences in the frequencies of LPMC T_EM_ and T_EMRA_ in unvaccinated elderly than in adults. Oral Ty21a immunization resulted in alterations of the levels of these memory subsets in the local mucosa which differed between adults and the elderly. Age-associated differences in the responses of CD4+ and CD8+ T cells have been previously reported in response to vaccination against viral (e.g., influenza, yellow fever), bacterial (e.g., *Streptococcus* pneumonia) and parasitic (e.g., *Plasmodium falciparum*) infectious diseases [11, 57–60]. Our studies confirm age-associated differences in CD4+ and CD8+ responses that correlate with lower vaccination efficacy in older adults [8, 61, 62]. However, most studies were performed using PBMC. In contrast, our data showing that age affects immunity following oral immunization with an attenuated oral vaccine to a major bacterial pathogen extend these findings by providing direct evidence that aging influences not only the systemic, but also mucosal vaccine-induced responses. These results suggest that oral Ty21a vaccination of older individuals may not be as efficacious as younger adults, due, at least in part, to the observed reductions in Th17 effectors, an important component of immunity in mucosal surfaces. Interestingly, our data indicate that CD4+ T_EM_ and T_RM_ vaccine-induced responses are the major subsets in the TI mucosa impacted by aging, both in terms of quantity (frequencies) and characteristics of the responses. For example, oral Ty21a immunization elicits significantly lower multifunctional CD4+ T_EM_ (CD107a, IFNγ, IL-17A, IL-2, TNFα and MIP1β) responses in the elderly than younger adults. Similar observations were recorded in the production of IL-17A and IL-2 from elderly CD4+ T_RM_ subsets. Lower levels of *S*. Typhi-responses (e.g., IL-17A and IL-2) were also observed in TI-LPMC CD8+ T_RM_ and CD103-CD8+ T cells obtained from elderly subjects. While most *S*. Typhi-specific responses were lower in elderly than adult volunteers, this did not seem to be due to an intrinsic factor of cells in the elderly, since they were capable of responding to stimulation. For example, following α-CD3/CD28 stimulation, we observed that all cytokines evaluated (IFNγ, IL-17A, IL-2 and TNFα) produced by CD4+ T_RM_ were similar in elderly and adults. One caveat in our study is that the median age difference between the two age groups (e.g., < 60 and ≥ 60 yrs.) is 11 years in the unvaccinated group and 15 years in Ty21a-vaccinees. This is due to the fact that routine indicated colonoscopies in health adults are medically indicated after the age of 45. It is likely that T_RM_ obtained from terminal tissues of younger adults (18–45 yrs) might have shown even larger differential responses between elderly and young adult volunteers. Taken together, these data provide evidence that aging decreased Ty21a vaccine-induced responses not only systemically but also in the gut, the preferred site of entry of *S.* Typhi. Of note, these impaired responses are not due to LPMC T cells not being able to produce cytokines since the CD3 activation pathway and the main costimulatory pathway (CD28) appear to be functioning similarly in adults and the elderly. Future studies are needed to elucidate the mechanisms operating in the local mucosa that are hampering vaccine-induced responses.

T_RM_ are now considered essential components of immunological memory as numerous reports have demonstrated the important roles of T_RM_ in mediating protective immunity [17, 18]. Human intestinal T_RM_ are established early in childhood and increase in frequency throughout adulthood [63]. In the elderly population, T_RM_ frequencies are high and stable in the intestine where they are mostly found in the lamina propria and intraepithelial lymphocyte compartments (IEL) [63]. Here, we extend these finding by showing that LPMC CD4+ T_RM_ subsets are influenced by aging, particularly in terms of higher frequencies of CD103- CD4+ T_RM_ but lower effector responses, especially IL-17A and IL-2, in elderly volunteers following Ty21a immunization. On the other hand, CD8+ T_RM_ and CD103- CD8+ T cells showed no differences in the number of cells present in the mucosa, but also exhibited significantly reduced IL-17A and IL-2 responses in elderly volunteers. While it is difficult to assess the lifespan and tissue retention of human T_RM_ over time in vivo, studies of the age-associated changes in human T_RM_ have been performed in other tissues (e.g., lungs) to determine T_RM_ responses to pathogens, especially viruses. Of note, our observations of age-associated changes in the intestine following Ty21a vaccination are consistent with those reported in the literature in other tissues. For example, a recent study investigated how lung CD8+ T_RM_ are affected by aging and how they respond in vitro to exposure to influenza and SARS-CoV-2 [64]. Interestingly, the authors reported that lung CD8+ T_M_ are very susceptible to age-associated attrition and showed that CD8+ CD103+ CD69+ T_RM_ influenza-specific cells declined with age while CD8+ CD69- CD103- T frequencies increases [64]. In contrast, unlike exposure to influenza, exposure of lung CD8+ T_M_ cells (including T_RM_) to SARS-CoV-2 did not elicit the induction of pro-inflammatory cytokines (including Type I, II and III interferon) irrespective of the age of the volunteers [64]. Of note, these volunteers were naïve to SARS-CoV-2 and presumably would not have any SARS-CoV-2-specific T_RM_ in the lungs. Taken together, these data suggest that T_RM_ subsets are retained in the local mucosa as we age, but their characteristics, functions and numbers of some its subsets are affected. Future studies are needed to address age-associated changes in the T cell compartment in specific segments of the gastrointestinal and respiratory tracts, subset-specific dynamics and their characteristics in order to elucidate T_RM_’s potential as targets for future vaccines.

Recent data have suggested that impaired vaccine-induced immunity in the elderly is associated with inflammaging and immunosenescence [65, 66]. HCMV is considered a major contributor to inflammaging, which is generally asymptomatic in healthy individuals [67]. About 25–90% of the world wide population is HCMV seropositive, with higher prevalence in older adults [68, 69]. The establishment of a latent infection by HCMV is a common event likely correlated to immunosenescence by increasing the levels of highly differentiated effector memory cells in the CD8+ and CD4 + T-cell pools [70]. It has been reported that the presence of increased numbers of specific T_reg_ and T follicular helper cells during HCMV infection limits the efficacy of influenza vaccination in older people, likely through rendering less capable to provide help to B cells when faced with new antigens [71]. In contrast, it has been reported that HCMV infection does not appear to influence the rate of change of various lymphocytes (e.g., T and NK cells) [34]. In our study, we observed that similar percentages in all groups were seropositive for HCMV. Moreover, when we compared *S.* Typhi-specific IL-17A responses between HCMV positive and negative individuals in both Ty21a vaccinated and unvaccinated volunteers, we observed no significant differences between these two groups. Thus, HCMV seropositivity did not appear to be a major determinant of the age-related dissimilarities observed in *S*. Typhi-specific vaccine-induced responses. However, these observations could be due to the relatively low number of volunteers, health status and age range of our volunteers (47–73 yrs.). Further studies will need to address HCMV-specific memory T_RM_ responses and their correlation with the TCR repertoire and clonal expansion of T cell subsets induced by Ty21a immunization.

## Conclusion

Similar to infant vaccination schedules, the elderly population would benefit from highly effective bacterial vaccines, particularly those targeted to common and serious infections caused by pathogens such as *S. pneumoniae, E. coli, S. aureus, C. difficile, K. pneumoniae* and Mtb. These vaccines might be more effective if tailored to optimally elicit immunity at the site of infection (e.g., mucosal surfaces of the respiratory and gastrointestinal tracts) and utilized the protective potential of T_RM_ to express the appropriate immune correlated of protection against these pathogens. Here, we provide the first evidence of age-associated differences of the induction of tissue resident CD4+ and CD8+ T_RM_ following Ty21a immunization. These results contribute novel insights in our understanding of the effect of aging on immune cells in human tissues and the generation of mucosal immunity in the elderly following immunization with oral attenuated bacteria.

## Methods

### Volunteers, immunization and sample collection

Individuals (aged 49–74 yrs) undergoing routine colonoscopy were enrolled from the Baltimore-Washington metropolitan area and University of Maryland, Baltimore campus. Volunteers who have no previous history of typhoid fever and were not vaccinated with the attenuated oral Ty21a typhoid vaccine were assigned to each of two groups. Four recommended doses of Ty21a (Vivotif enteric-coated capsules; Crucell, Bern, Switzerland) [72] were administered to the first group (Adult (< 60 yrs) *n* = 10; Elderly (≥60 yrs) *n* = 8) but not to the control group (Adult *n* = 21; Elderly *n* = 8). Blood samples were collected 14–21 days before colonoscopy (pre-immunization) for the purpose of generating autologous EBV-B cell lines to be used in the *S.* Typhi-specific assays. The day of the colonoscopy (day 0; 14 to 21 days post vaccination), large capacity forceps were used to obtain terminal ileum biopsies [26]. PBMC were isolated immediately after blood draws by density gradient centrifugation and cryopreserved in liquid nitrogen following standard techniques [73].

### Isolation of lamina propria mononuclear cells (LPMC) from terminal ileum biopsies

TI-LPMC was freshly isolated as previously described [26, 74–76]. Briefly, after collection of biopsies from volunteers undergoing routine colonoscopy, tissues were treated with HBSS (without CaCl_2_, MgCl_2_, MgSO_4_; Gibco, Carlsbad, CA) and EDTA (10 mM; Ambion, Grand Island, NY) and were vigorously shaken for 45 min to remove IEL. Next, the biopsies were digested enzymatically with collagenase D (100 μg/mL; Roche, Indianapolis, IN) and DNase I (10 μg/mL; Affymetrix, Cleveland, OH) for 45 mins followed by homogenization using a Bullet Blender homogenizer (Next Advance Inc., Averill, NY) to extract LPMC. LPMC were either stained immediately for immunophenotyping by flow cytometry or stimulated overnight.

### Target cell preparation and *S*. Typhi infection

Autologous Epstein-Barr virus (EBV)-transformed lymphoblastoid cell line (EBV-B cells) were generated from each participant’s pre-immunization PBMC as previously described [73, 77]. Target cells were then infected with wt-*S.* Typhi strain ISP1820 at a MOI of 7:1 as previously described [25]. Infected target cells were then gamma-irradiated (6000 rad) before ex vivo LPMC stimulation. To confirm *S*. Typhi infection, target cells were stained with anti-*Salmonella* common structural Ag (CSA-1, Kierkegaard and Perry, Gaithersburg, MD) and analyzed by flow cytometry as previously described [73, 77].

### Stimulation of terminal ileum LPMC

Freshly isolated TI-LPMC were used as effector cells as previously described [25, 26]. Briefly, LPMC were co-cultured, respectively, with either non-infected or *S*. Typhi-infected EBV-B (MOI of 7:1). LPMC cultured with media only or in the presence of α-CD3/CD28 (Life technologies, Grand Island, NY) were used as negative and positive controls, respectively. After 2 h, 0.5 μl of Golgi Stop (Monensin, BD) and 0.5 μl Golgi Plug (Brefeldin A, BD) were added and cultures continued overnight at 37 °C in 5% CO_2_.

### Surface and intracellular staining

Following overnight stimulation, TI LPMC were stained for flow cytometry analysis as previously described [26]. Briefly, LPMC were stained for live/dead discrimination (YEVID) (Invitrogen, Carlsbad, CA) and then the Fc receptors were blocked using human immunoglobulin (3 μg/mL; Sigma). This was followed by surface staining. Briefly, for the LPMC T_RM_ panel, cells were stained at 4 °C for 30 min with fluorescently labeled monoclonal antibodies (mAbs) directed to CD13-Pacific Orange (conjugated in-house), CD19-BV570 (HIB19, Biolegend, San Diego, CA), CD3-BV650 (OKT3, Biolegend), CD4-PE-Cy5 (RPA-T4, BD), CD8-PerCP-Cy5.5 (SK1, BD), CD45RA-biotin (HI100, BD), CD62L-APC-A780 (DREG-56, eBioscience, San Diego, CA), and CD103-FITC (Ber-ACT8, BD). After a wash, cells were stained with streptavidin (SAV)-Qdot800 (Invitrogen) at 4 °C for 30 min. Cells were then fixed and permeabilized using IC fixation and permeabilization buffers (8222/8333, eBioscience). This was followed by staining at 4 °C overnight with mAbs directed to interleukin (IL)-17A-BV421 (BL168, Biolegend), interferon (IFN)-γ-PE-Cy7 (B27, BD), tumor necrosis factor (TNF)-α-Alexa 700 (MAb11, BD), CD69-ECD (TP1.55.3, Beckman Coulter, Danvers, MA), and IL-2-BV605 (MQ1-17H12, Biolegend). For the LPMC T_EM_ panel, cells were stained with fluorescently labeled mAbs directed to CD19-BV570 (HIB19, BioLegend), CD13-Pacific Orange (conjugated in-house), CD3-BV650 (OKT3, BioLegend), CD4-PE-Cy5 (RPA-T4, BD), CD8-PerCP-Cy5.5 (SK1, BD), CD45RA-biotin (HI100, BD), CD62L-APC-A780 (DREG-56, eBioscience) and integrin α4β7-A647 (ACT1; conjugated in-house) at 4 °C for 30 min. As for the T_RM_ panel, cells were stained with streptavidin (SAV)-Qdot800 (Invitrogen) at 4 °C for 30 min. Cells were then fixed and permeabilized using IC fixation and permeabilization buffers (8222/8333, eBioscience). This was followed by staining (4 °C overnight) with mAbs directed to IL-17A-BV421 (BL168, BioLegend), IFNγ-PE-Cy7 (B27, BD), TNFα-Alexa 700 (MAb11, BD), and CD69-ECD (TP1.55.3, Beckman Coulter, Danvers, MA, USA), IL-2-BV605 (MQ1-17H12, BioLegend), and MIP1β-PE (IC271P, R&D Systems). After staining, cells were stored in 1% paraformaldehyde at 4 °C until data collection. Data were collected using a customized LSRII flow cytometer (BD) and then analyzed using the WinList version 7 (Verity Software House, Topsham, ME) software package. *S.* Typhi-specific responses were expressed as net percentage of positive cells (background after stimulation with uninfected cells were subtracted from values obtained with *S.* Typhi–infected targets). The FCOM function of WinList was used to determine *S.* Typhi–specific MF responses in TI LPMC. Flow cytometry experiments were performed at the Flow Cytometry and Mass Cytometry Core Facility of the University of Maryland School of Medicine Center for Innovative Biomedical Resources (CIBR), Baltimore, Maryland.

### HCMV ELISA

The volunteers’ sera were kept frozen at -20 °C until testing for HCMV specific IgG by enzyme-linked immunoassay (GenWay Biotech Inc., San Diego, CA) which was performed according to the manufacturer’s instructions. A corrected absorbance value of 0.25 was considered positive.

### Statistical analysis

Data were analyzed using the statistical software GraphPad Prism™ version 7 (Graphpad, San Diego, CA, USA). Statistical differences in median values between two groups were determined using Mann–Whitney tests. Correlations (Age versus frequencies of T_RM_ and T_EM_) were evaluated using Pearson’s correlation tests. Based on a recent recommendation by the American Statistical Association (ASA), particularly when analyzing data sets with relatively low numbers of volunteers, we also indicated trends in expression of markers or cytokine responses where appropriate using a *P* ≤ 0.1 [32, 33].

## Supplementary Information

**Additional file 1: Figure S1.** Gating strategy and age-dependent frequency of T cells obtained from terminal ileum biopsies. (**A**) Gating Strategy to define CD4+ and CD8+ T memory subsets and tissue resident subsets in terminal ileum LPMC. (**B**) Frequencies of TI-LPMC CD3, CD4 and CD8 were measured and compared between adults (< 60 yrs.; A) and elderly (≥60 yrs.; E) volunteers obtained from Ty21a-vaccinated (red bars) and unvaccinated volunteers (black bars). Significant differences indicated (*P < 0.05). Trends to exhibit significance are indicated by their *p*-value. Horizontal black bars represent median values.

**Additional file 2: Figure S2.** Frequencies of systemic and mucosal CD4+ and CD8+ T_EM_ expressing CD69 or CD103 in adults and elderly volunteers following Ty21a immunization. The percentages of cells expressing CD69 or CD103 were determined in **(A)** LPMC CD4+ T_EM_, and **(B)** LPMC CD8+ T_EM_ following Ty21a vaccination. Significant differences indicated (*P < 0.05; **P < 0.005). Trends to exhibit significance are indicated by their *p*-values. Horizontal black bars represent median values.

**Additional file 3.**

**Additional file 4.**

**Additional file 5.**

## Data Availability

The datasets supporting the findings of this study are available within the article and its Supplementary Information files.

## Abbreviations

PBMC: Peripheral blood mononuclear cells
LPMC: Lamina propria mononuclear cells
T_RM_: Tissue resident memory T cell
EBV-B: Epstein-Barr virus (EBV)-transformed lymphoblastoid B cells
MF: *S.* Typhi-specific multifunctional cells
S: *S.* Typhi-specific single cytokine producing cells
TI: Terminal ileum
T_EM_: T-effector/memory- (CD62L- CD45RA-)
T_CM_: T-central/memory - (CD62L+ CD45RA-)
T_EMRA_: T_EM_-CD45RA+ − (CD62L- CD45RA+)
LP: Lamina propria
