# Supplementary figures and images for: Age-dependency of terminal ileum tissue resident memory T cell responsiveness profiles to S. Typhi following oral Ty21a immunization in humans
Source: Immun Ageing. 2021 Apr 19;18:19. doi: 10.1186/s12979-021-00227-y (PMC8053564; doi:10.1186/s12979-021-00227-y)

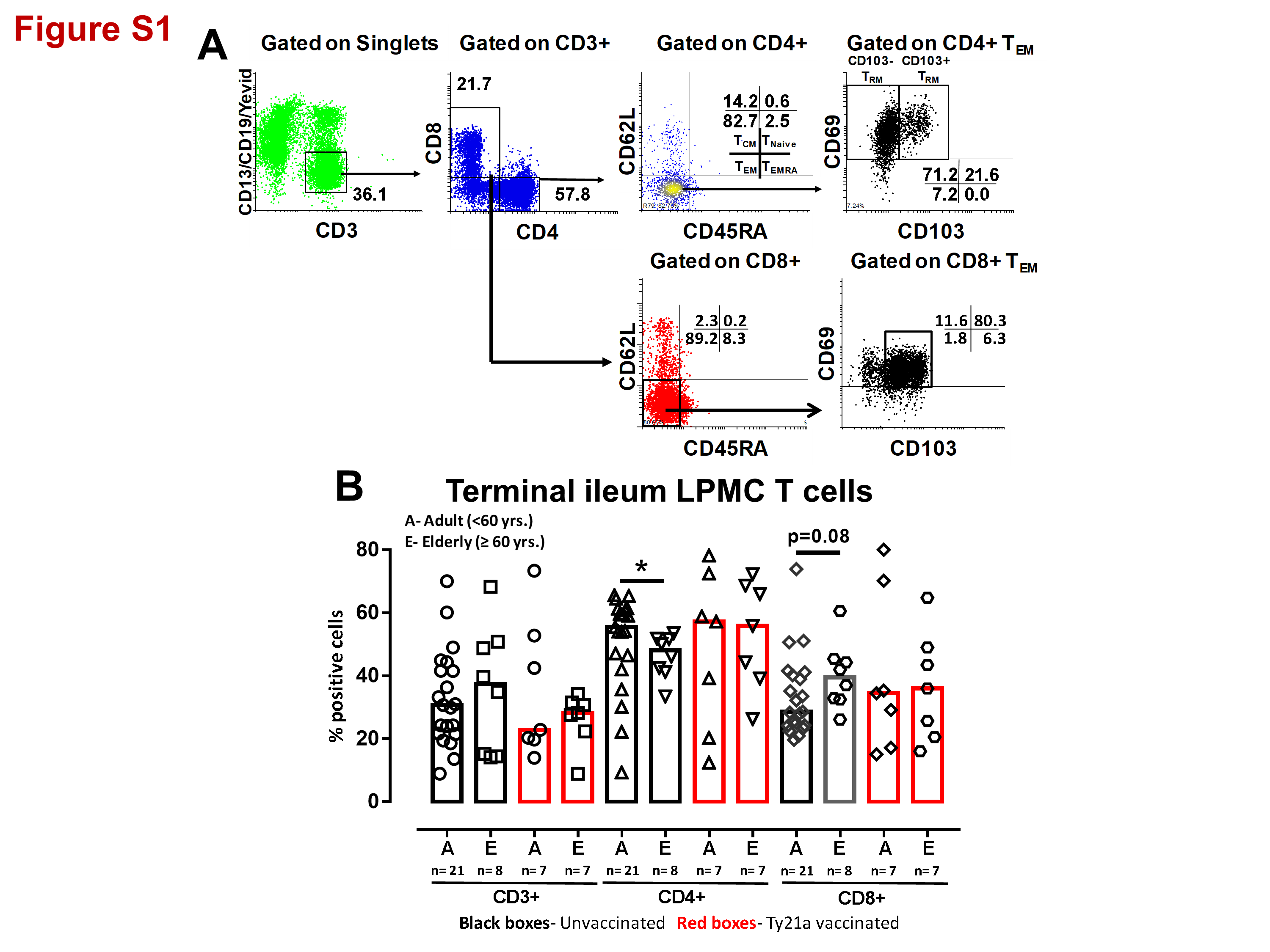

Supplement: Supplementary file 1 — Additional file 1: Figure S1. Gating strategy and age-dependent frequency of T cells obtained from terminal ileum biopsies. (A) Gating Strategy to define CD4+ and CD8+ T memory subsets and tissue resident subsets in terminal ileum LPMC. (B) Frequencies of TI-LPMC CD3, CD4 and CD8 were measured and compared between adults (< 60 yrs.; A) and elderly (≥60 yrs.; E) volunteers obtained from Ty21a-vaccinated (red bars) and unvaccinated volunteers (black bars). Significant differences indicated (*P < 0.05). Trends to exhibit significance are indicated by their p-value. Horizontal black bars represent median values. [file 12979_2021_227_MOESM1_ESM.tif]

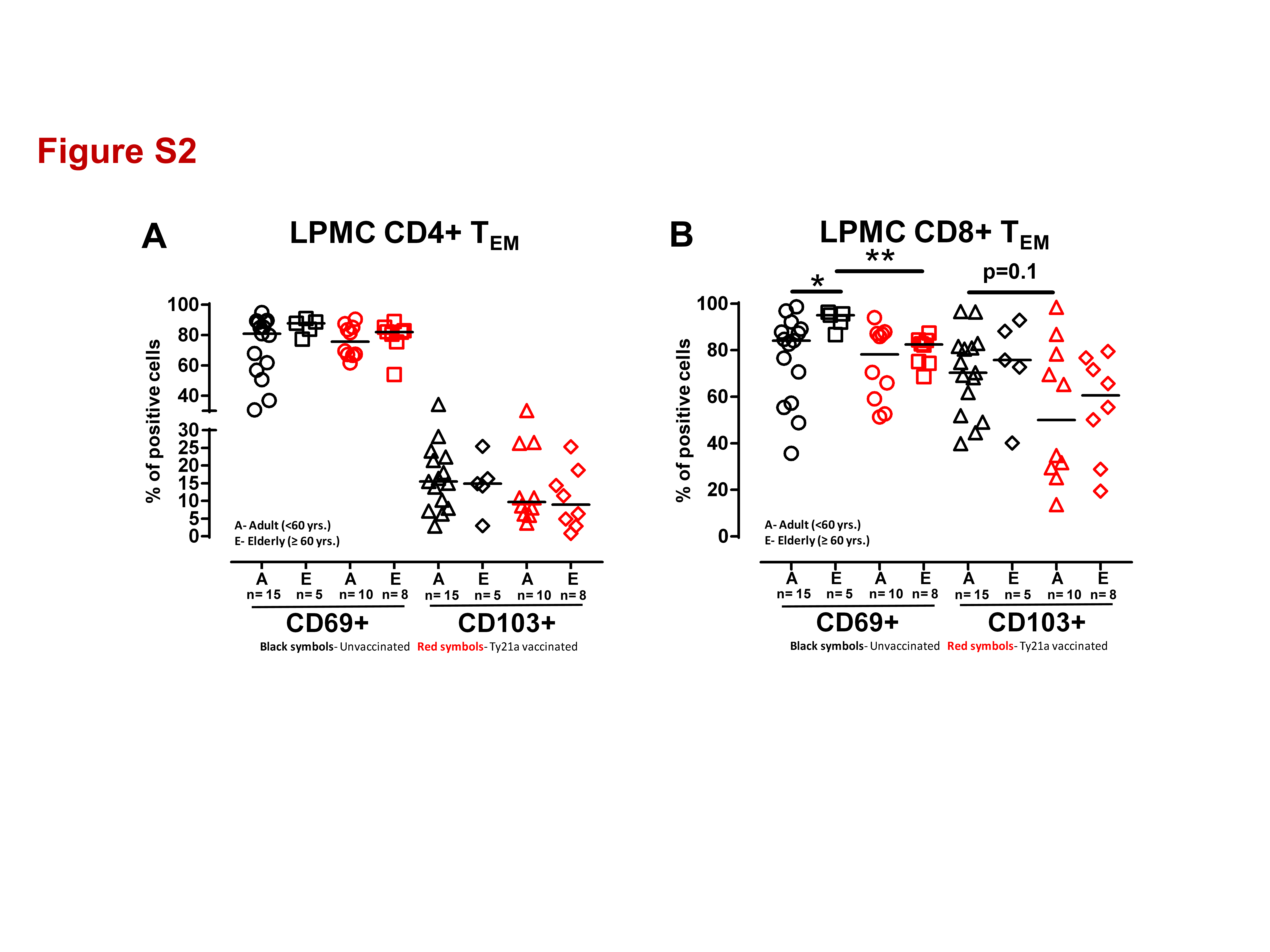

Supplement: Supplementary file 2 — Additional file 2: Figure S2. Frequencies of systemic and mucosal CD4+ and CD8+ TEM expressing CD69 or CD103 in adults and elderly volunteers following Ty21a immunization. The percentages of cells expressing CD69 or CD103 were determined in (A) LPMC CD4+ TEM, and (B) LPMC CD8+ TEM following Ty21a vaccination. Significant differences indicated (*P < 0.05; **P < 0.005). Trends to exhibit significance are indicated by their p-values. Horizontal black bars represent median values. [file 12979_2021_227_MOESM2_ESM.tif]

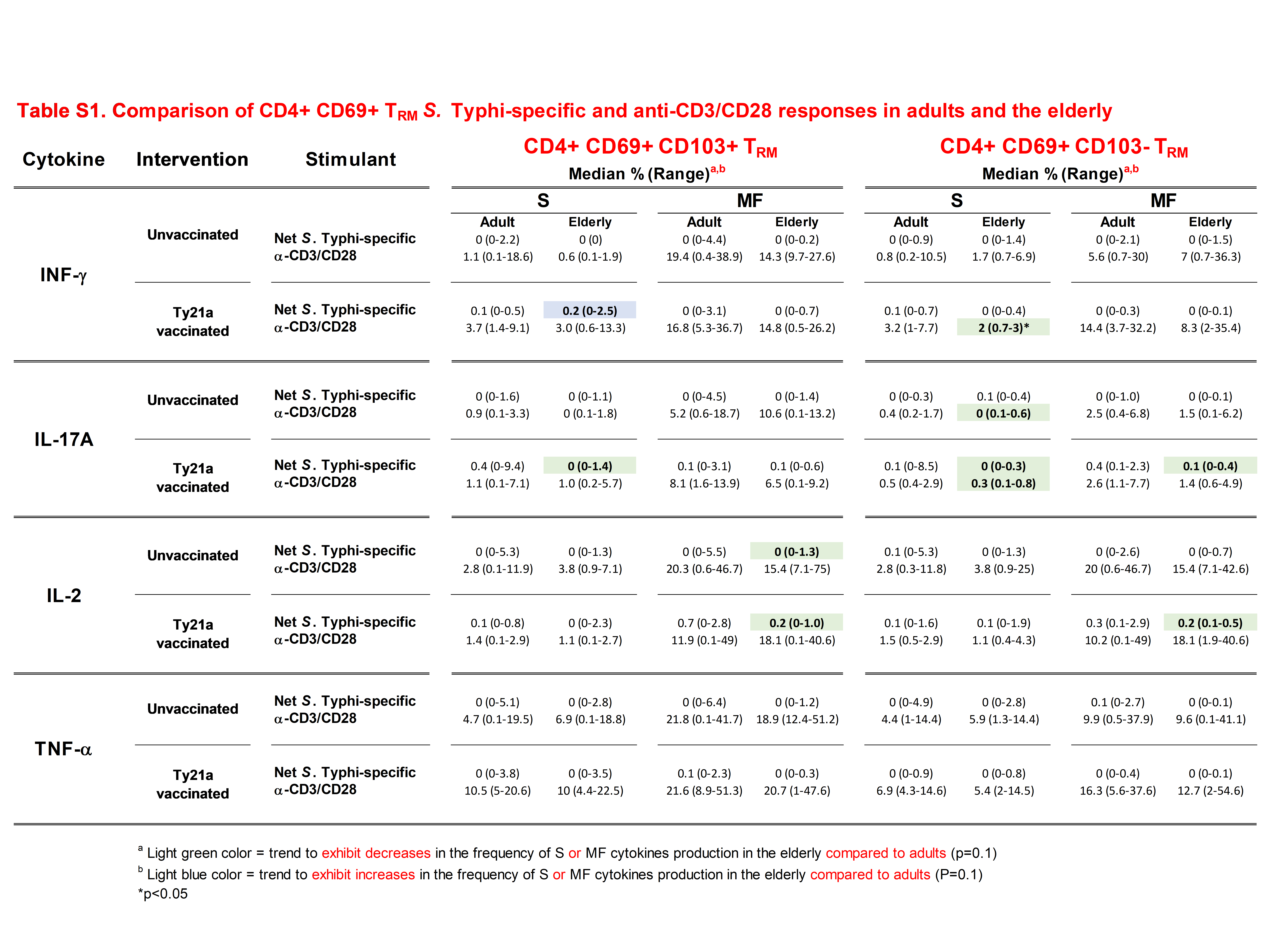

Supplement: Supplementary file 3 — Additional file 3. [file 12979_2021_227_MOESM3_ESM.tif]

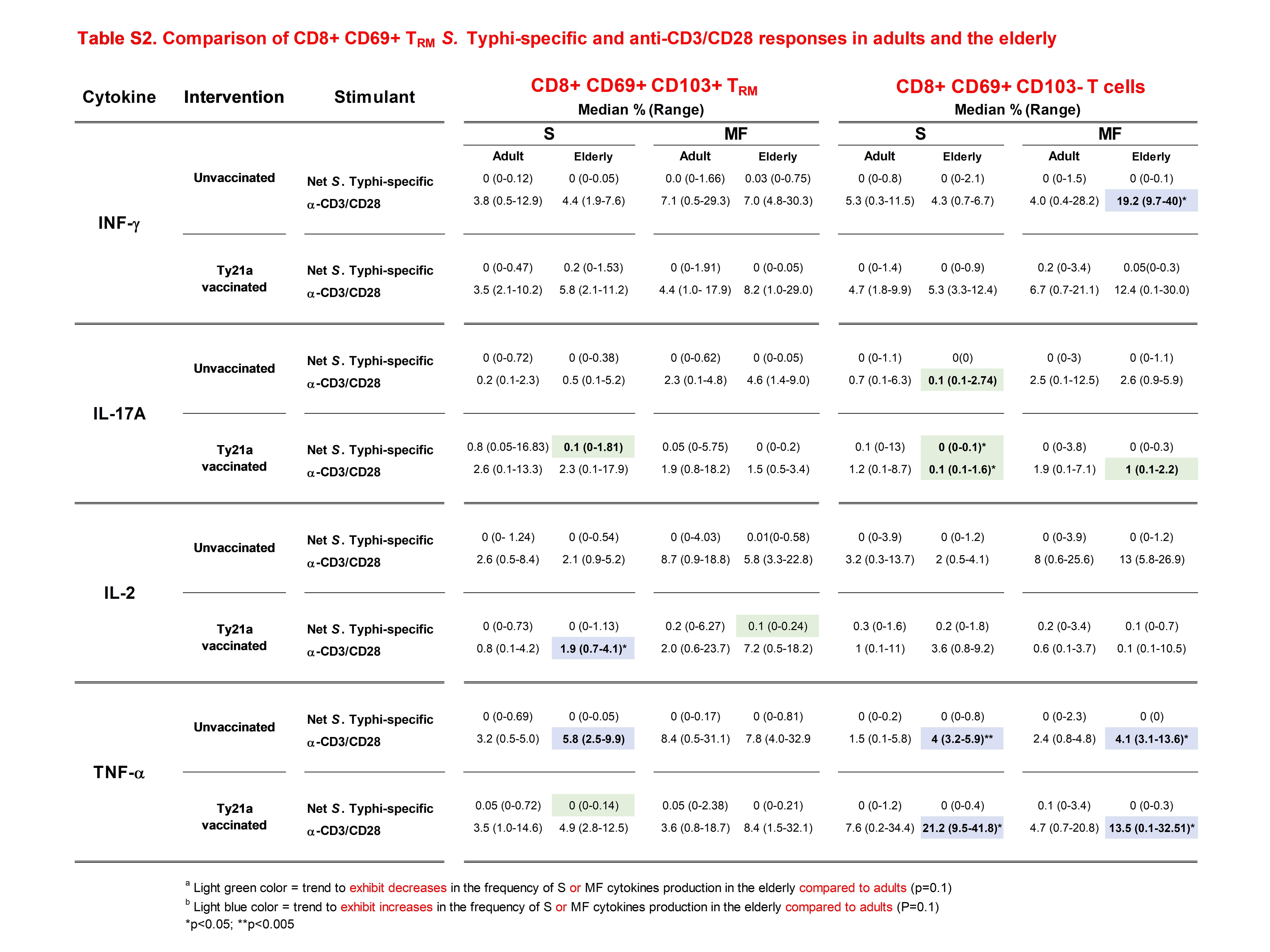

Supplement: Supplementary file 4 — Additional file 4. [file 12979_2021_227_MOESM4_ESM.tif]

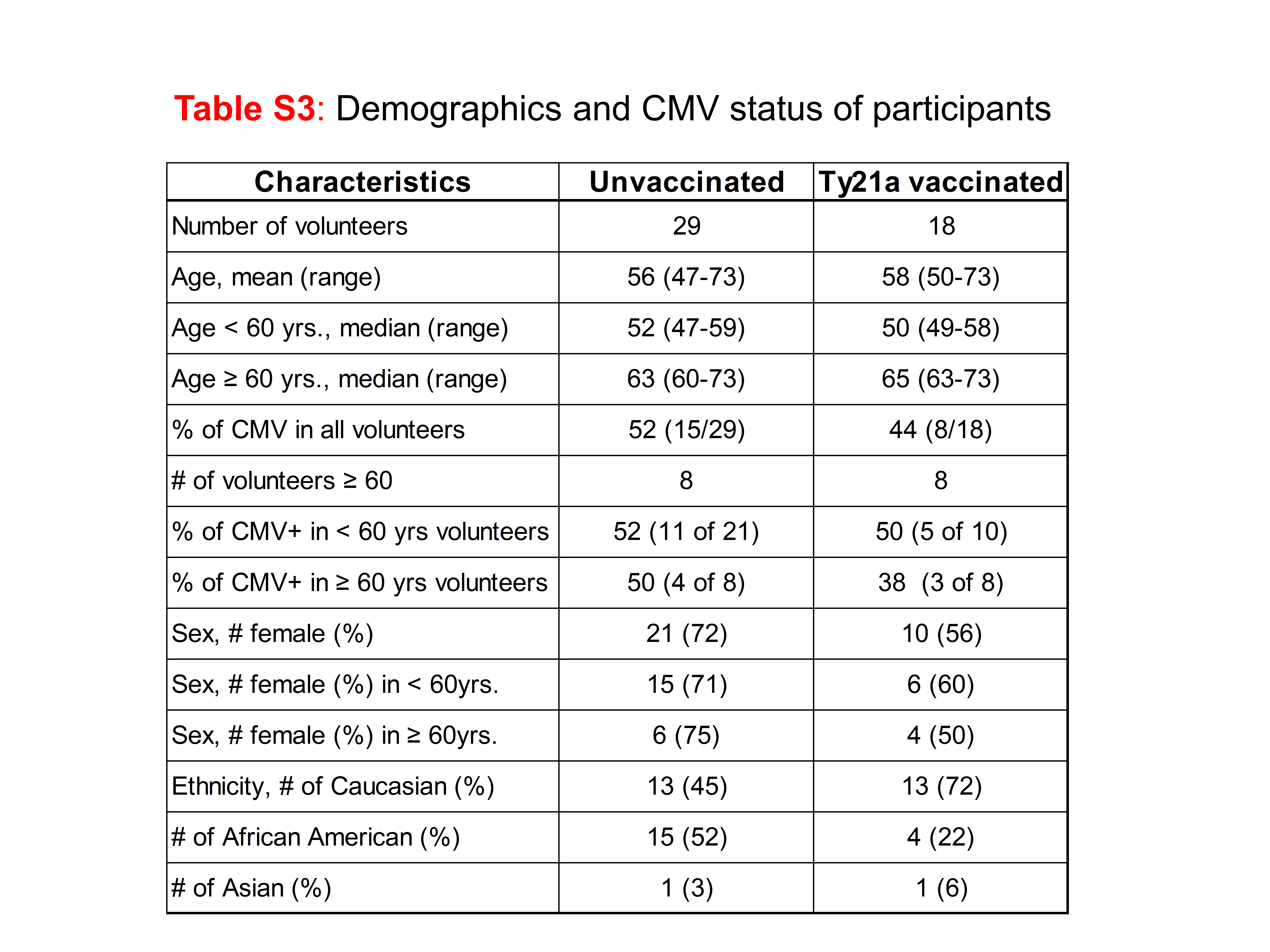

Supplement: Supplementary file 5 — Additional file 5. [file 12979_2021_227_MOESM5_ESM.tif]
